# Radiocopper in BCMA-targeted immunotheranostics of myeloma

**DOI:** 10.7150/thno.134397

**Published:** 2026-07-29

**Authors:** Martin Ullrich, Kristof Zarschler, Manja Kubeil, Markus Laube, Jens Pietzsch, Birgit Belter

**Affiliations:** 1 Helmholtz-Zentrum Dresden-Rossendorf, Institute of Radiopharmaceutical Cancer Research, Department of Radiopharmaceutical and Chemical Biology, Bautzner Landstrasse 400, D-01328 Dresden, Germany.; 2 Helmholtz-Zentrum Dresden-Rossendorf, Institute of Radiopharmaceutical Cancer Research, Department of Medicinal Radiochemistry, Bautzner Landstrasse 400, D-01328 Dresden, Germany.; 3 Helmholtz-Zentrum Dresden-Rossendorf, Institute of Resource Ecology, Department of Radiation Research on Biological Systems, Bautzner Landstrasse 400, D-01328 Dresden, Germany.; 4 Technische Universität Dresden, School of Science, Faculty of Chemistry and Food Chemistry, D-01062 Dresden, Germany.

**Keywords:** multiple myeloma, B cell maturation antigen, small animal PET, small animal SPECT, xenograft model, copper-64, copper-67, bispidine chelators

## Abstract

**Background:**

The theranostic potential of copper-64 (PET imaging) and copper-67 (SPECT imaging and therapy) is increasingly recognized. This work investigates the performance of this ‘true’ matched pair in a preclinical radioimmunotheranostic setting that utilizes experimental monoclonal antibodies (mAbs) directed against B cell maturation antigen (BCMA), a transmembrane glycoprotein that has emerged as a critical target for the treatment of multiple myeloma (MM).

**Methods:**

The commercially available BCMA-directed mAbs MAB193 and Vicky-1 were modified with the bispidine N_2_py_4_ enabling chelation of radiocopper, and their binding affinity was determined using surface plasmon resonance and flow cytometry. Pharmacokinetics and tumor uptake of the copper-64-labeled mAbs were determined in mice bearing subcutaneous myeloma xenografts, using PET. The best-performing mAb was included in a pilot study on therapeutic use with copper-67 in U266 myeloma-bearing mice, involving dose monitoring and dose predictions for humans based on quantitative SPECT.

**Results:**

After bioconjugation, both [^64^Cu]Cu-N_2_py_4_-MAB193 and [^64^Cu]Cu-N_2_py_4_-Vicky-1 maintained low nanomolar BCMA binding affinity. In PET imaging, [^64^Cu]Cu-N_2_py_4_-MAB193 showed the highest uptake in U266 myeloma xenografts and the lowest normal tissue background, e.g., achieving a tumor-to-muscle contrast of 14.9 within 48 h. [^67^Cu]Cu-N_2_py_4_-MAB193 delivered absorbed doses up to 1.26 Gy/MBq in U266 myeloma xenografts and reduced the tumor mass at total doses above 52 Gy. Predicted human effective doses were below 35.3 µSv/MBq for immunoPET and 162 µSv/MBq for radioimmunotherapy/immunoSPECT.

**Conclusion:**

The fundamental efficacy of copper-64 and copper-67 in BCMA-targeted immunotheranostics of MM promises both precise PET-based dose planning as well as radioimmunotherapy in combination with highly sensitive SPECT-based dose monitoring, as demonstrated by the theranostic capabilities of [^64^Cu/^67^Cu]Cu-N_2_py_4_-MAB193 in tumor-bearing mice. The results provide a strong incentive for incorporating the *CopperNostics* approach into the further development of BCMA-targeted radioimmunotheranostic agents, including precise tailoring of their pharmacokinetic properties to the physical half-life of copper-67.

## Introduction

As radionuclides of the same element, copper-64 (PET imaging, physical half-life 12.7 h) and copper-67 (β‾ therapy and SPECT imaging, physical half-life 61.8 h) are increasingly recognized as a ‘true’ matched pair with potential for medical use in cancer theranostics 1, 2. The physical half-life of copper-64 is sufficiently consistent with the slow pharmacokinetics of monoclonal antibodies (mAbs) and thus offers advantages over shorter-lived radionuclides in immunoPET imaging 3. The mean energies of β‾ particles emitted by copper-67 (150 keV) provide the same radiotherapeutic efficacy as those of lutetium-177 (147 keV) 4, 5. Concomitantly, a high relative abundance of γ photons enables high-sensitivity SPECT imaging 6, 7. Commercial production via accelerator-based photonuclear reaction yields copper-67 in sufficient quantities and purity, opening up realistic prospects for expanding the portfolio of therapeutic radiopharmaceuticals that can be produced independent of nuclear reactors 7.

To date, the theranostic value of copper-64-labeled agents addressing critical cancer targets has been demonstrated *in vivo* for numerous classes of compounds, including small molecules, peptides, antibody fragments, mAbs, and nanoparticles 1, 7. In contrast, the theranostic potential of copper-67 has been demonstrated primarily for peptide receptor radionuclide therapies targeting critical cell surface receptors in cancer, most frequently somatostatin type 2 receptors (SSTR2) 1, 5, 7. For a limited range of cancer-relevant antigens, several preclinical studies have demonstrated the therapeutic efficacy of copper-67-labeled radioimmunoconjugates, e.g., mAbs targeting human leucocyte antigen DR10, human epidermal growth factor receptor 2 (HER-2), and mucin 1 (MUC1) 6, 8-12. The promising therapeutic effects reported therein suggest expanding the potential applications of copper-67-labeled mAbs to other cancer-relevant antigens and warrant a thorough analysis of their therapeutic dose delivery.

B cell maturation antigen (BCMA, also known as TNFRSF17 or CD269), a transmembrane glycoprotein belonging to the tumor necrosis factor receptor superfamily, has emerged as a critical target for innovative immunotherapies of multiple myeloma (MM), a malignancy characterized by the uncontrolled proliferation of plasma cells within the bone marrow 13-18. A fraction of BCMA is cleaved from the surface of the myeloma cells by γ-secretase, resulting in the release of soluble BCMA (sBCMA) into the bloodstream 19, 20. BCMA-directed therapeutics, encompassing chimeric antigen receptor T cells, bispecific T cell engagers, and antibody-drug conjugates, have undergone testing in MM patients 21, 22. However, despite high objective response rates, relapses do occur, and tumor cells develop resistance to current BCMA-targeted immunotherapies 23, challenging further development and improvement of treatment strategies.

Myeloma cells display inherent sensitivity to radiation, with the most extensive clinical experience gained from external beam radiation therapy and from peptide receptor radionuclide therapy targeting the chemokine receptor CXCR4 24-26. Given the clinically proven efficacy of the existing non-radioactive BCMA-directed treatments, several radiolabeled agents for BCMA-specific targeting of myeloma cells have been successfully tested in preclinical settings so far. These developments were based on peptides, single-domain antibodies (sdAbs), mAbs, and one mAb-nanoparticle conjugate, and involved radiolabeling with gallium-68, copper-64, zirconium-89, fluorine-18, lutetium-177, and iodine-131 27-32. Recently, a gallium-68-labeled nanobody-based BCMA-tracer has been successfully tested in PET imaging of MM in a prospective first-in-human trial (NCT06717113) 33. These promising results provide a strong incentive to expand BCMA-directed radioimmunotheranostic approaches.

For the immunotheranostic application of radiocopper, chelators that form stable complexes and enable fast radiolabeling of the biological vector molecules under mild reaction conditions are of particular interest. Due to their preorganized geometry, hexadentate bispidines (3,7-diazabicyclo[3.3.1]nonane derivatives) are particularly suitable for the selective complex formation with Cu^II^ radionuclides, resulting in metal complexes with high thermodynamic stability and kinetic inertness 34-38.

This work investigates the potential and practical applicability of copper-64 and copper-67 in radioimmunotheranostics within an experimental setting that utilizes mAbs targeting BCMA in models of human myeloma. The preclinical study reports on (i) the radiochemical and binding properties of two human BCMA-selective mAbs, **MAB193** and **Vicky-1**, labeled with radiocopper trough complexation with a covalently linked bispidine (N_2_py_4_), (ii) the distribution and pharmacokinetic properties of **[^64^Cu]Cu-N_2_py_4_-MAB193** and **[^64^Cu]Cu-N_2_py_4_-Vicky-1** in mice measured by quantitative PET imaging, including their performance in visualizing subcutaneous U266 and L363 myeloma xenografts, and (iii) radiation doses from treatment with **[^67^Cu]Cu-N_2_py_4_-MAB193**, estimated based on quantitative SPECT imaging in U266 tumor-bearing mice. A critical discussion on therapeutic dose delivery by the copper-67-labeled anti-BCMA bispidine-mAb conjugate is provided.

## Materials and Methods

### Chemicals, solvents, and antibodies

Chemicals and solvents were purchased from common vendors (Merck, Darmstadt, Germany; Thermo Scientific, Waltham, MA, USA; VWR, Radnor, PA, USA) and used without further purification. Deuterated solvents were purchased from Deutero. Milli-Q® water (resistivity 18.2 MΩ × cm at 25°C) was used for high performance liquid chromatography (HPLC) purification and radiochemical experiments.

Anti-human BCMA mAbs were purchased from commercial suppliers: **MAB193** (rat monoclonal IgG_2A_ Clone 335004, Cat. No. MAB193, Bio-Techne, Minneapolis, MN, USA); **Vicky-1** (rat monoclonal IgG_1_ Clone Vicky-1, Cat. No. ALX-804-151, ENZO Life Sciences, Lausen, Switzerland); **E6D7B** (rabbit monoclonal IgG Clone E6D7B, Cat. No. 88183, Cell Signaling Technology Europe, Leiden, Netherlands), and **IgG_2A_-ITC** (rat monoclonal IgG_2A_ isotype control, Cat. No. MAB006, Bio-Techne).

The anti-human BCMA sdAb **269A37948** (patent WO-2018028647-A1 39) was produced by cloning the nucleotide sequence (GenBank: LQ 790725.1) into a pET-28b vector (Merck) followed by functional gene expression and purification of the recombinant protein as described recently 37, 40.

### Synthesis of bispidine-mAb conjugates

The functionalized hexadentate bispidine **N_2_py_4_-benzyl-isothiocyanate (N_2_py_4_-Bn-NCS)** was synthesized, purified, and underwent quality control as described previously 35, 37, 41.

The anti-human BCMA mAbs **MAB193** and **Vicky-1** were reconstituted in freshly prepared sodium bicarbonate saline buffer (50 mM NaHCO_3_, 150 mM NaCl, pH 8.5) and functionalized with a 10-fold molar excess of **N_2_py_4_-Bn-NCS,** M = 741.9 g/mol, c = 10 nmol/µL DMSO) at 37 °C for 4 h with gentle shaking. The bispidine-mAb conjugates **N_2_py_4_-MAB193** and **N_2_py_4_-Vicky-1** were purified by size-exclusion chromatography using ZebaSpin Desalting Columns (7K MWCO, 5 mL, Thermo Scientific, Waltham, MA, USA) with elution in ammonium acetate buffer (0.2 M NH_4_OAc, pH 6) as well as spin filtration using Amicon Ultra centrifugal filters (30 K MWCO, 0.5 mL, Merck). Protein concentrations were determined using the DC Protein Assay (Cat. No. 5000116, Bio-Rad Laboratories, Hercules, CA, USA). Bispidine-mAb conjugates underwent quality control using matrix-assisted laser desorption ionization time-of-flight (MALDI-TOF) mass spectrometry as described in the *Supporting information* and were stored at 4 °C until further use.

To ensure batch-to-batch consistency, reaction conditions for bioconjugation (molar ratio of antibody-to-chelator, buffer composition, pH, reaction volume, time, and temperature) were kept constant, and freshly prepared chelator batches as well as freshly reconstituted antibody lyophilizates were used at all times.

### Radiolabeling of bispidine-mAb conjugates

Copper-64 and copper-67 were produced with a TR-FLEX cyclotron (Advanced Cyclotron Systems Inc., Richmond, BC, Canada) by a ^64^Ni(p,n)^64^Cu and a ^70^Zn(p,α)^67^Cu nuclear reaction, respectively, as described previously.^42^-^44^ For radiolabeling of bispidine-mAb conjugates, a substance-to-[^64^Cu]CuCl_2_ ratio of 1 nmol : 100 MBq and a substance-to-[^67^Cu]CuCl_2_ ratio of 1 nmol : 25 MBq was maintained. The reactions were set up in ammonium acetate buffer (0.2 M NH_4_OAc, pH 6) and incubated at 37 °C for 30 min with gentle shaking. The radiolabeled antibody-to-[^64^Cu/^67^Cu]CuCl_2_ ratio was assessed by radio-TLC using iTLC-SG and 0.05 M aq. EDTA (pH 5.5) and reported as radiochemical purity (%). Radiolabeled bispidine-mAb conjugates underwent quality control using radio-thin layer chromatography (radio-TLC) and denaturing sodium dodecyl sulfate-polyacrylamide gel electrophoresis (SDS-PAGE) as described in the *Supporting information*.

### Cells

The human myeloma cell lines U266 (Cat.No. ACC 9) and L363 (Cat.No. ACC 49) were purchased from the DSMZ (Braunschweig, Germany) and cultured in Roswell Park Memorial Institute 1640 Medium (Thermo Scientific, Cat No. 61870036,) supplemented with 10% (v/v) fetal bovine serum (Merck) and 1 U/mL penicillin/streptomycin (Thermo Scientific, Cat No. 11548876,). The human melanoma cell line A375 (Cat.No. CRL-1619) was purchased from ATCC (LGC, Wesel, Germany) and cultured in Dulbecco’s modified Eagle medium (Thermo Scientific, Cat No. 12077549) supplemented with 10% (v/v) fetal bovine serum and 1 U/mL penicillin/streptomycin. The cells were cultivated in a CO_2_ incubator (37 °C, 5% (v/v) CO_2_, 95% (v/v) humidity).

### Tumor xenograft models

Animal experiments were carried out according to the guidelines of the German Regulations for Animal Welfare. The protocols were approved by the local Ethical Committee for Animal Experiments (license DD24.1-5131/449/49 and 25-5131/562/52). 8 to 12 weeks old female Rj:NMRI-*Foxn1*^nu/nu^ mice (Janvier Labs, Saint Berthevin Cedex, France) were subcutaneously injected with 5 × 10^6^ U266 or L363 cells in 100 µL isotonic NaCl (aq.) containing 50% (v/v) VitroGel® Hydrogel Matrix (TheWell Bioscience, Monmouth Junction, NJ, USA), or with 5 × 10^6^ A375 cells in 100 µL isotonic NaCl (aq.) into the right hind leg. Tumor size was monitored by caliper measurements and tumor volume was calculated using the formula *V*_Tumor_ = π/6 × (length × width^2^). Tumor-bearing mice were included in the experiments when tumors reached a volume of 150 mm^3^. After the final experiment, anesthetized animals were sacrificed under desflurane anesthetic (Baxter, Deerfield, IL; USA) using CO_2_ inhalation and cervical dislocation.

### Gamma secretase inhibitor treatment

To reduce the release of sBCMA from myeloma xenografts into the blood stream, U266 and L363 tumor-bearing mice were treated with a γ secretase inhibitor (GSI, Crenigacestat LY3039478, MedChemExpress, Monmouth Junction, NJ, USA), dissolved in corn oil containing 10% (v/v) DMSO. Animals received four consecutive GSI treatments in 24 h-intervals, each with a dose of 2.5 mg/kg administered in 150 µL through oral gavage.

### Immunohistochemistry

Formalin-fixed and paraffin-embedded sections of tumor xenografts (5 µm) were prepared according to standard procedures. Antigen-demasking was performed in sodium citrate buffer (10 mM, pH 6.0, 95°C, 20 min), followed by blocking of endogenous peroxidase and biotin using Biotin Blocking System (Agilent Technologies, Santa Clara, CA, USA, Cat. No. X0590) and blocking of non-specific binding sites with gelatin blocker (50 mM Tris, pH 8.0, 60 mM NaCl, 0.3% (v/v) gelatin, 2% (w/v) bovine serum albumin, 3% (w/v) milk powder, 0.5% (v/v) Tween20). Samples were incubated with primary antibody (polyclonal rabbit anti-human BCMA/TNFRSF17, Abcam, Cambridge, UK, Cat. No. ab5972) or mAb isotype control (normal Rabbit IgG, Abcam, Cat. No. ab27478) at 4°C overnight, followed by secondary antibody (biotinylated goat anti-rabbit antibody, Cat. No. 111-065-003, Jackson ImmunoResearch, Baltimore Pike, PA, USA) at room temperature for 1 h. Sections were incubated with ExtrAvidin® peroxidase for 30 min (Cat. No. E2886, Merck), stained with AEC substrate (Cat. No. 551015, BD Biosciences, Franklin Lakes, NJ, USA) and counterstained with Mayer’s Hematoxylin.

### Enzyme-linked immunosorbent assay (ELISA)

For quantification of BCMA concentrations in tumor explants as well as in blood serum (sBCMA) tumor-bearing mice were anesthetized, blood was collected by cardiac puncture followed by immediate cervical dislocation. Blood was allowed to coagulate for 20 min at room temperature, followed by centrifugation at 3000 × g for 5 min at 4 °C. Clear serum was transferred into fresh microtubes and stored at -80°C until further use. Tumors were excised, cut into halves, and pieces of 2-4 mm from the central part of the tumor were quickly frozen in liquid nitrogen. Tissue pieces were transferred to a 2-mL-microtube containing 600 µL of lysis buffer (150 mM NaCl, 50 mM Tris pH 7.5, 1% NP-40, freshly added protease inhibitor cocktail tablets, Cat. No. 04693159001, Roche, Basel, Switzerland), and two steel balls, and disrupted in a TissueLyser device (Qiagen, Venlo, The Netherlands) at 25 Hz at room temperature for 3 min. Samples were treated with ultrasound for 15 s, stored on ice for 20 min, followed by centrifugation at 16.000 × g at 4 °C for 10 min. Protein content was determined using a BCA protein assay kit (Fisher Scientific) according to the manufacturer’s instructions. All samples were diluted to 1 mg/mL total protein in lysis buffer and stored at -80 °C.

The amount of BCMA in whole-cell lysates and sBCMA in mouse blood serum was determined using the human BCMA/TNFRSF17 DuoSet ELISA (Cat. No. DY193, Bio-Techne). Samples were thawed on ice and diluted 1:40 (tumors, finally 25 µg of protein/mL) or 1:40-1:200 (serum) in Reagent Diluent. ELISA was performed according to the manufacturer’s instructions.

### Positron emission tomography

Small-animal positron emission tomography (PET) was performed using the nanoScan PET/CT (Mediso Medical Imaging Systems, Budapest, Hungary). Elevated serum concentrations of myeloma-related sBCMA were reduced by daily treatment with GSI (-1 d prior to 3 d after injection of the radiolabeled mAb). Each mouse received between 10 and 15 MBq of the radiolabeled mAb (eq. to 0.11-0.17 nmol) delivered in isotonic NaCl (aq.) via intravenous injection through a tail vein catheter within the initial 30 s after scan start. A series of PET scans were performed at defined time points (with scan durations) after injection of the radiolabeled mAb: 1 h (0-1 h), 5 h (4.5- 5.5 h), 24 h (23-25 h), and 48 h (47-49 h). With each scan, a corresponding CT image was captured and used for anatomical referencing and attenuation correction. Binning and time framing were performed as reported previously 45. Images were reconstructed using the Tera-Tomo™ three-dimensional (3D) algorithm with a voxel size of 0.4 mm applying corrections for attenuation, scattering, and decay.

### Saturation binding on tumor cryosections

Cryosections (10 µm) were prepared from tumor explants and mounted onto SuperFrost® plus glass slides (Thermo Scientific). Tissue sections were warmed to room temperature, incubated in Dulbecco’s phosphate-buffered saline for 15 min, air-dried, and incubated with **[^64^Cu]Cu-N_2_py_4_-MAB193** serially diluted in binding buffer (Dulbecco’s phosphate-buffered saline containing 2.5% (w/v) bovine serum albumin) to final concentrations increasing from 0.025 to 50 nM for 1.5 h at room temperature. Non-specific binding was measured in presence of a BCMA-specific competitor (sdAb **269A37948**) at final concentrations increasing from 0.25 to 500 nM (10-fold molar excess for each concentration). Tissue sections were washed twice in binding buffer for 5 min, dipped in distilled water, and air-dried. BAS-SR phosphoimaging plates (Fujifilm, Tokyo, Japan) were exposed to tissue sections and standards (increasing molar amounts of the radiolabeled mAb) for 45 min and imaged using an Amersham Typhoon^TM^ FLA 9500 (Cytiva). Photostimulated luminescence (PSL) intensities were extracted from radioluminographic images using AIDA 5.1 (Elysia-raytest, Straubenhardt, Germany). Intensities per tissue section were converted into molar amounts of bound radiolabeled mAb using the standard series. Binding constants were calculated from non-linear regression analysis using the ‘one-site specific binding’ model implemented in Prism 11 (GraphPad Software, Boston, MA, USA). Binding capacities (*B*_max_) were expressed as molar amount per mm^3^ of tissue.

### Radioimmunotherapy and single-photon emission computed tomography

Elevated serum concentrations of myeloma-related sBCMA were reduced by daily treatment with GSI (-1 d prior to 3 d after injection of the radiolabeled mAb). U266 tumor-bearing mice (*n* = 3) were treated with **[^67^Cu]Cu-N_2_py_4_-MAB193** (*A*_m_ = 70 MBq/nmol), each with a different initial activity dose of 25 MBq (eq. to 0.36 nmol), 50 MBq (eq. to 0.71 nmol), or 80 MBq (eq. to 1.14 nmol) administered in 0.2 mL of isotonic NaCl (aq.) through injection into a tail vein. Quantitative small-animal single-photon emission computed tomography (SPECT) was performed using the nanoScan® SPECT/CT (Mediso Medical Imaging Systems) equipped with the APT56 UHE aperture. Emission of photons was recorded and binned within the 20% energy windows of the 93 and 185 keV photopeaks 5. Images were acquired within the following time windows after injection of the radiolabeled mAb (with corresponding scan times): 0.9-1.1 h (30 min), 25-27 h (40 min), 47-49 h (50 min), and 5 d (60 min). With each SPECT scan, a corresponding CT image was recorded and used for anatomical referencing and attenuation correction. Images were reconstructed using the Tera-Tomo™ 3D algorithm with a voxel size of 0.23 mm applying corrections for attenuation, scattering, and decay.

### Analyses of PET and SPECT images

PET and SPECT images were post-processed and analyzed using Rover version 3.0.77h (Abx, Radeberg, Germany) and displayed as maximum intensity projections with common scaling over indicated time points. Details on the extraction of tissue-specific uptake values from PET images (region-averaged standardized uptake values, SUVmean) and SPECT images (% of initial activity/mL) are provided in the *Supporting information (*Figure S 1). Tissue-specific time courses of uptake values (decay-corrected and normalized to the initial activity dose) were analyzed by non-linear regression using the ‘two-phase decay’ and ‘one-phase decay’ models implemented in Prism 1 (GraphPad Software), with extrapolated uptake values approaching a lower limit constrained to 0. Total uptake in the liver, kidneys, and spleen [% of initial activity/organ] was extrapolated from the locoregional uptake values using a realistic dosimetry model for 30-g-mice 46. The physical density of tissues was assumed to be 1 g/cm^3^.

### Dose calculations

All doses were calculated based on the quantitative analysis of PET or SPECT images. For estimating the absorbed energy doses [Gy] resulting from uptake of radioimmunoconjugates in myeloma xenografts, the time courses of uptake values were transformed into time courses of effective activity concentrations by considering the physical half-life of the specific radionuclide involved [% of initial activity/mL]. The time-cumulated activity per mL of tissue was calculated from areas under effective time-activity concentration curves [% of initial activity dose/mL × d] and further converted into the total time-cumulated activity in the tumor-specific volume [MBq × h]. The mean absorbed tissue dose *D*_T_ in tumors was calculated using the sphere model implemented in Olinda 2.2.3 (Hermes Medical Solutions AB, Stockholm, Sweden).

For human dose predictions, the tissue-specific pharmacokinetic profiles of radioimmunoconjugates in mice were transformed into human time scales and human organ masses using allometric scaling as described elsewhere 47. Projected cumulated activity in human organs was determined separately for female and male subjects, and projected human absorbed doses [µGy/MBq] and effective doses [µSv/MBq] were calculated using the ICRP 89 Adult Female (60 kg) and ICRP 89 Adult Male (73 g) models implemented in Olinda 2.2.3 (Hermes Medical Solutions) as described previously 5.

### Statistical analysis

Statistical analysis was performed using Prism 10 (GraphPad Software). Unless stated otherwise, data are presented as means with standard deviation and *n* represents the number of experiments or animals. Unless stated otherwise, significance of differences was tested using ANOVA with Sidak *post-hoc* test.

## Results

### Binding affinity of anti-BCMA mAbs

The three commercially available anti-human BCMA mAbs **MAB193** (rat IgG_2A_), **Vicky-1** (rat IgG_1_), and **E6D7B** (rabbit IgG) were the starting point for investigating an experimental BCMA-directed radioimmunotheranostic approach against MM. The three mAbs were initially characterized in its pristine form regarding their binding affinity towards recombinant human BCMA using surface plasmon resonance (SPR) interaction analysis as well as to native, cellular BCMA using flow cytometry employing the human cancer cell lines U266 and L363 (characterized by *high* and *low* cellular BCMA levels, respectively). Both **MAB193** and **Vicky-1** showed binding affinities towards BCMA with calculated equilibrium dissociation constants (*K*_d_) in the subnanomolar (SPR) and nanomolar (flow cytometry) range as well as species selectivity for human BCMA (Table 1 and *Supporting information*, Figure S 2, Figure S 3, Figure S 5). **E6D7B** showed no binding to recombinant human or murine BCMA and was therefore excluded from further investigations.

### Generation of radiocopper-labeled bispidine-mAb conjugates

Bioconjugation of the BCMA-affine mAbs with the amine-reactive bispidine derivative **N_2_py_4_-Bn-NCS** resulted in the bispidine-mAb conjugates **N_2_py_4_-MAB193** and **N_2_py_4_-Vicky-1**. For control experiments, the non-specific mAb isotype control **IgG_2A_-ITC** was also equipped with the bispidine chelator resulting in **N_2_py_4_-IgG_2A_-ITC**. The three bispidine-mAb conjugates showed degrees of conjugation between 0.5 and 1.0 determined by MALDI-TOF mass spectrometry (*Supporting information,* Figure S 4).

After covalent modification with the bispidine chelator, the abilities of both **N_2_py_4_-MAB193** and **N_2_py_4_-Vicky-1** for recognizing BCMA on U266 cells remained unchanged (*Supporting information,* Figure S 5 E-F), providing the rationale to further investigate the radiocopper-labeled bispidine-mAb conjugates. Radiolabeling with copper-64 provided preparations of **[^64^Cu]Cu-N_2_py_4_-MAB193**,** [^64^Cu]Cu-N_2_py_4_-Vicky-1,** and **[^64^Cu]Cu-N_2_py_4_-IgG_2A_-ITC** with molar activities between 50 and 80 MBq/nmol and radiochemical purities of > 99% determined by radio-TLC (*Supporting information*, Figure S 6).

### BCMA concentrations in subcutaneous human myeloma xenografts

**Figure 1:**
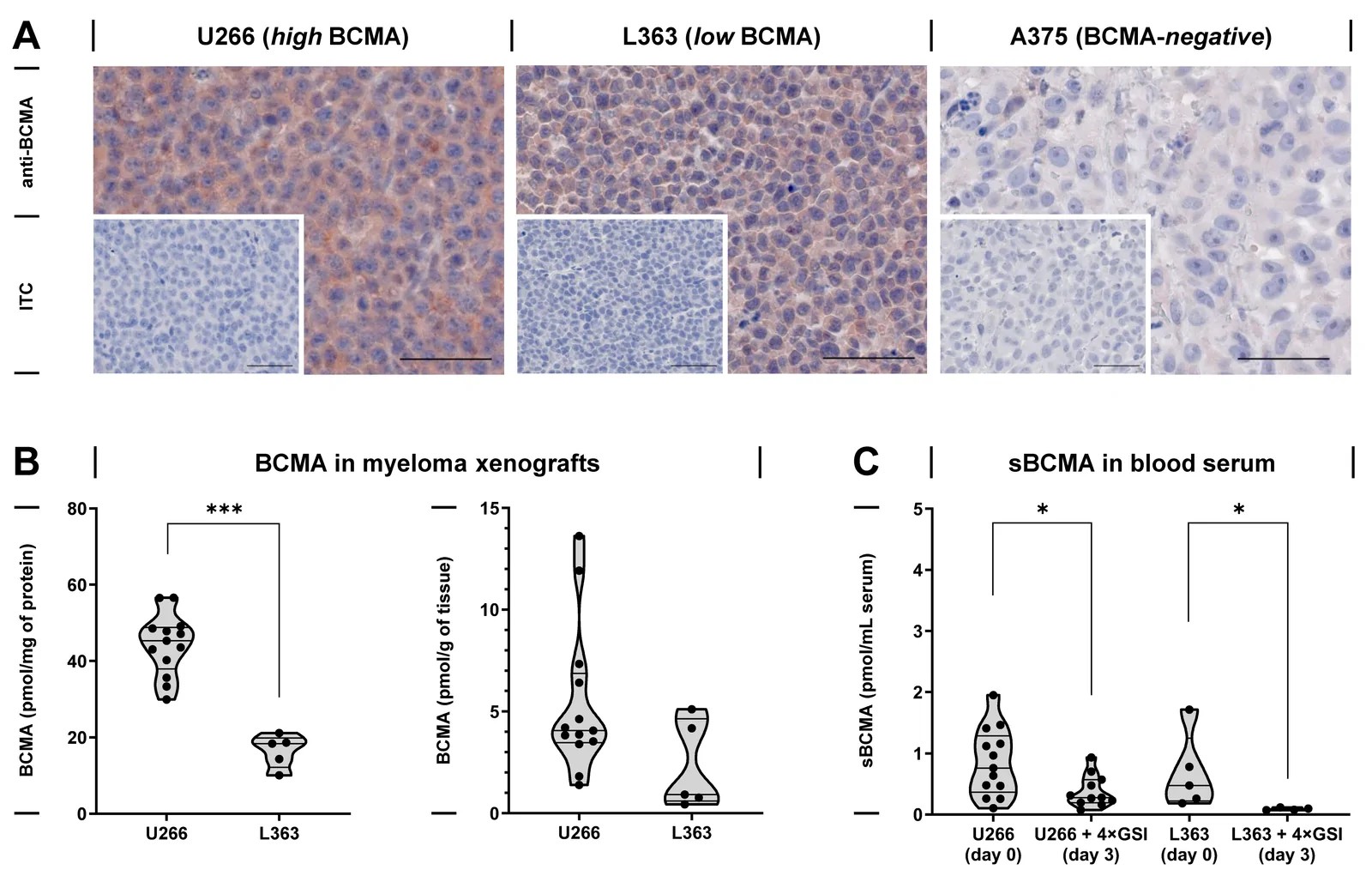
BCMA and sBCMA in mice bearing subcutaneous human myeloma xenografts; (A) Immunohistochemical staining of U266, L363 and A375 tumor microsections; insets: negative controls incubated with a mAb isotype control; scale bars: 50 µm; (B) Concentrations of BCMA in myeloma homogenates measured by ELISA; (left) concentrations normalized to protein content, (right) concentrations normalized to tissue mass; (C) Concentrations of tumor-derived sBCMA in the blood serum measured by ELISA; reduction of the initial concentrations (day 0) after four daily treatments (day 0-3) with a γ-secretase inhibitor (GSI); significance of differences determined using Mann-Whitney test: * *p* < 0.05*** *p* < 0.001.

Prior to *in vivo* testing of radiocopper-labeled anti-BCMA mAbs in mice bearing human myeloma xenografts, BCMA concentrations in subcutaneous tumors as well as sBCMA concentrations in the blood serum were characterized. Immunohistochemistry showed positive staining for BCMA in tissue sections from U266 and L363 myeloma xenografts, but not in A375 melanoma xenografts serving as negative controls (Figure 1 A). Quantitative analysis of BCMA in tissue lysates by ELISA showed 2.7-fold higher concentrations in U266 compared to L363 tumors (44.4 ± 8.08 *versus* 16.5 ± 4.35 pmol/mg of protein, respectively; Figure 1 B). Serum samples obtained from mice bearing U266 and L363 myeloma xenografts showed the release of sBCMA into the blood (0.85 ± 0.55 and 0.68 ± 0.62 pmol/mL, respectively; Figure 1 C). Treatment with GSI for 4 d effectively reduced the concentration of sBCMA in the blood serum obtained from both U266 and L363 tumor-bearing mice (to 0.38 ± 0.26 and 0.09 ± 0.02 pmol/mL, respectively). These results provided the rationale to only include GSI-treated myeloma xenograft mice in the *in vivo* testing of radiocopper-labeled anti-BCMA mAbs in order to minimize their interaction with sBCMA in the blood.

### Distribution of copper-64-labeled anti-BCMA mAbs in tumor-bearing mice

The distribution of the radiocopper-labeled bispidine-mAb conjugates **[^64^Cu]Cu-N_2_py_4_-MAB193** and **[^64^Cu]Cu-N_2_py_4_-Vicky-1** was monitored by PET imaging for up to 48 h after injection in mice bearing subcutaneous BCMA-positive myeloma (U266 and L363) and BCMA-negative melanoma (A375) xenografts. Of note, **[^64^Cu]Cu-N_2_py_4_-Vicky-1** was excluded from further investigations *in vivo* due to its low tumor uptake and high liver background (*Supporting information*, Figure S 7 and Figure S 8).

**Figure 2:**
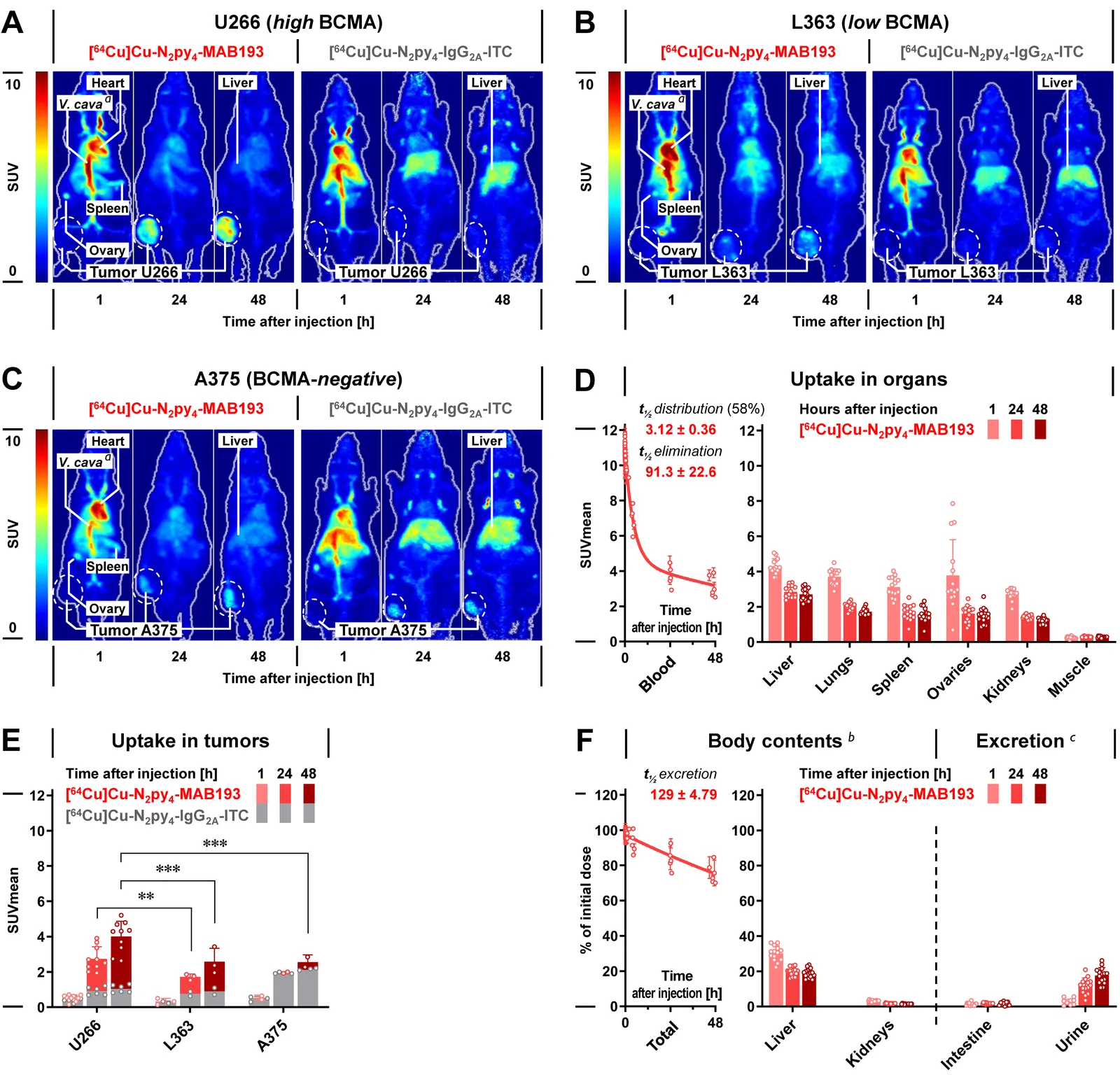
PET images and image-derived uptake values showing the distribution and tumor-specific accumulation of **[^64^Cu]Cu-N_2_py_4_-MAB193** depending on the different BCMA levels of subcutaneous U266, L363, and A375 tumor xenografts in nude mice; corresponding isotype control: **[^64^Cu]Cu-N_2_py_4_-IgG_2A_-ITC**; (A-C) PET maximum intensity projections; *^a^* signals from both *vena cava* and aorta; (D-E) Uptake values extracted from PET images; (F) Total activity contents and excretion: *^b^* extrapolated from uptake values using a dose model for 30 g-mice, *^c^* calculated through balancing activity contents in intestine, urinary bladder, and total body; see materials and methods for details; (SUVmean) region-averaged standardized uptake value, reference values measured *ex vivo* in tissue explants are included in the Supporting information, Figure S 8; means ± standard deviation; significance of differences: ** *p* < 0.01, *** *p* < 0.001.

In PET images,** [^64^Cu]Cu-N_2_py_4_-MAB193** enabled specific visual detection of both U266 and L363 tumors, unlike the corresponding isotype control **[^64^Cu]Cu-N_2_py_4_-IgG_2A_-ITC**, indicating that the accumulation of **[^64^Cu]Cu-N_2_py_4_-MAB193** in the myeloma xenografts was BCMA-specific (Figure 2 A, B). In A375 melanoma xenografts, both **[^64^Cu]Cu-N_2_py_4_-MAB193** and its corresponding isotype control** [^64^Cu]Cu-N_2_py_4_-IgG_2A_-ITC** showed low but comparable uptake, indicating that the accumulation of the two radiolabeled mAbs in the BCMA-negative tumor model was due to non-specific effects (Figure 2 C).

Quantitative analysis of PET images provided time courses of region-averaged standardized uptake values (SUVmean) for **[^64^Cu]Cu-N_2_py_4_-MAB193** in normal tissues and tumors (Figure 2 D, E). The radiolabeled mAb showed a biphasic pharmacokinetic profile in the blood with half-lives of 3.12 h for distribution (58% of the initial dose) and 87.8 h for elimination. Of note, blood kinetics of **[^64^Cu]Cu-N_2_py_4_-MAB193** were the same in γ-secretase inhibitor-treated myeloma-bearing mice and naïve A375 melanoma-bearing mice, ruling out effects from binding towards circulating sBCMA. After 48 h, 29% of the initial antibody concentration remained detectable in the blood.

Within 48 h, the uptake values decreased continuously in normal tissues of the liver, lungs, spleen, ovaries, kidneys, and muscles, while increasing to 4.01 and 2.59 in U266 and L363 tumors, respectively. The tumor-specific uptake of **[^64^Cu]Cu-N_2_py_4_-MAB193** corresponded to the higher BCMA levels of U266 compared to L363 cells. Consequently, the tumor-to-background ratios were higher in U266 compared to L363 myeloma-bearing mice (tumor-to-blood: 1.28 *versus* 0.68 [*p* < 0.001]; tumor-to-muscle: 14.9 *versus* 9.53 [*p* < 0.05], respectively). Radioluminographic analysis of cryosections prepared from tumor explants 24 h after **[^64^Cu]Cu-N_2_py_4_-MAB193** injection confirmed this trend (*Supporting information*, Figure S 9).

The distribution of the isotype control **[^64^Cu]Cu-N_2_py_4_-IgG_2A_-ITC** differed partially from that of **[^64^Cu]Cu-N_2_py_4_-MAB193**, most notably in liver uptake, remaining higher after 48 h (5.01 *versus* 2.72 [*p* < 0.001], respectively). Nevertheless, the U266 tumor-to-background ratios for **[^64^Cu]Cu-N_2_py_4_-IgG_2A_-ITC** remained well below those of **[^64^Cu]Cu-N_2_py_4_-MAB193** (tumor-to-blood: 0.45 *versus* 1.28 [*p* < 0.001]; tumor-to-muscle: 3.82 *versus* 14.9 [*p* < 0.001], respectively). A similar trend was observed in L363 tumor-bearing mice, indicating that the tumor contrast provided by **[^64^Cu]Cu-N_2_py_4_-MAB193** in the two myeloma xenografts models was BCMA-specific. SUVmean, tissue-to-background ratios, and statistical analyses are summarized in the *Supporting information* (Table S 2 and Table S 3).

The initially administered activity dose of **[^64^Cu]Cu-N_2_py_4_-MAB193** was excreted from the animals with a biological half-life of 129 h, predominantly via the renal pathway (Figure 2 F). After 48 h, 19% and 2.9% of the initial dose remained detectable in liver and kidneys, respectively.

The radiation doses predicted for immunoPET in humans using **[^64^Cu]Cu-N_2_py_4_-MAB193** were estimated based on quantitative PET data from U266 myeloma-bearing mice (*Supporting information,* Table S 4). Organs expected to receive the highest absorbed doses in the range between 66.2 and 138 µGy/MBq were spleen < ovaries < liver < heart wall. The predicted human effective doses were 35.3 µSv/MBq for women and 25.3 µSv/MBq for men, without taking tumor-specific accumulation into account.

### BCMA-specific binding of [^64^Cu]Cu-N_2_py_4_-MAB193 on tumor explants

**Figure 3:**
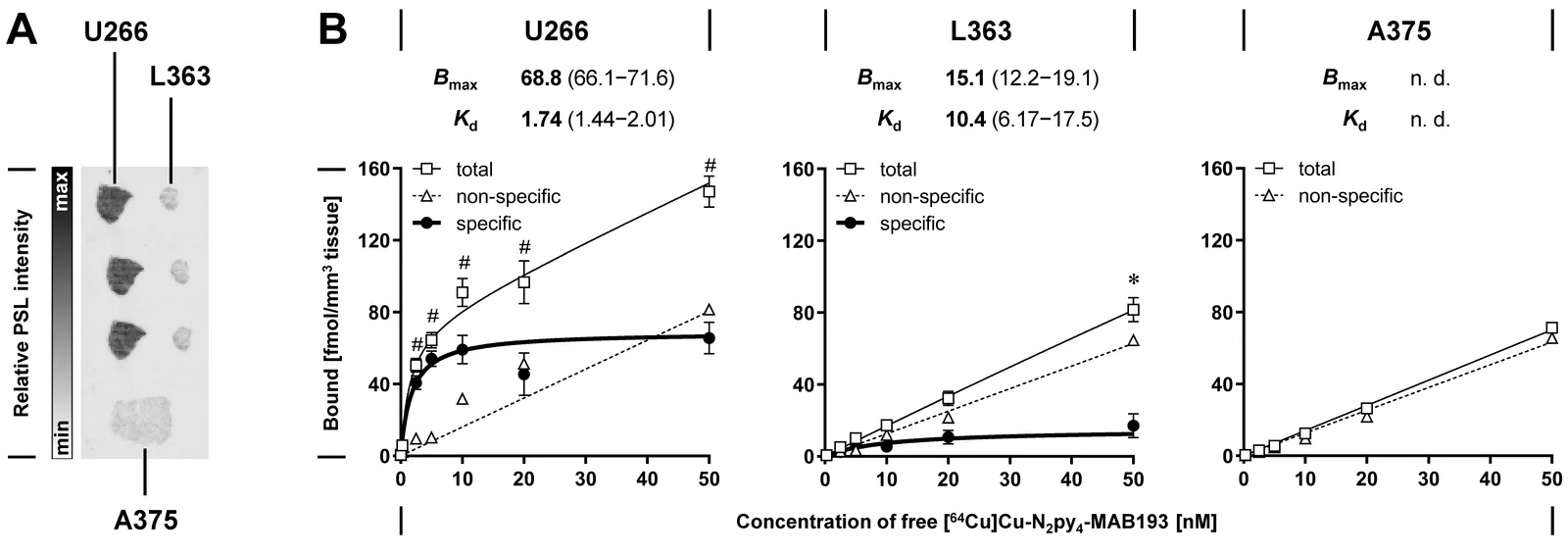
Binding of **[^64^Cu]Cu-N_2_py_4_-MAB193** to U266, L363, and A375 tumor cryosections *in vitro*; (A) Exemplary radioluminograms of tissue sections incubated with the radiolabeled mAb at a concentration of 5 nM; photostimulated luminescence (PSL) intensities; (B) Tumor-specific BCMA binding constants determined by quantitative analyses of radioluminograms (*n* = 3); non-specific binding was determined in presence of excess anti-human BCMA sdAb **269A37948**; binding constants were calculated using the non-linear regression model ‘one-site specific binding’; *B*_max_ values given in fmol/mm^3^ of tissue; *K*_d_ values given in nM; significance of differences compared to non-specific binding (ANOVA with Fisher’s LSD test): * *p* > 0.05, # *p* > 0.001; means with 68% confidence interval; (n.d.) not determined.

Whether the tumor-specific uptake of **[^64^Cu]Cu-N_2_py_4_-MAB193** in U266, L363, and A375 tumor xenografts *in vivo* corresponds to tumor-specific BCMA levels was further investigated by saturation binding on tumor cryosections *in vitro*. After incubation of the cryosections with **[^64^Cu]Cu-N_2_py_4_-MAB193**, quantitative analysis of radioluminograms showed equilibrium dissociation constants (*K*_d_) in the low nanomolar range and binding capacities (*B*_max_) that were five times higher on U266 compared to L363 tumor sections (Figure 3 A, B). Since the differences between total and non-specific binding reached statistical significance only for U266 and L363 tumor sections, binding towards A375 tumor sections was ascribed to non-specific effects. The *in vitro* binding capacities of U266, L363, and A375 cryosections for **[^64^Cu]Cu-N_2_py_4_-MAB193** are therefore consistent with the trends in tumor-specific uptake *in vivo*, indicating that the radiolabeled mAb is a BCMA-specific radiotracer.

### SPECT imaging and dose delivery of [^67^Cu]Cu-N_2_py_4_-MAB193 in tumor-bearing mice

**Figure 4:**
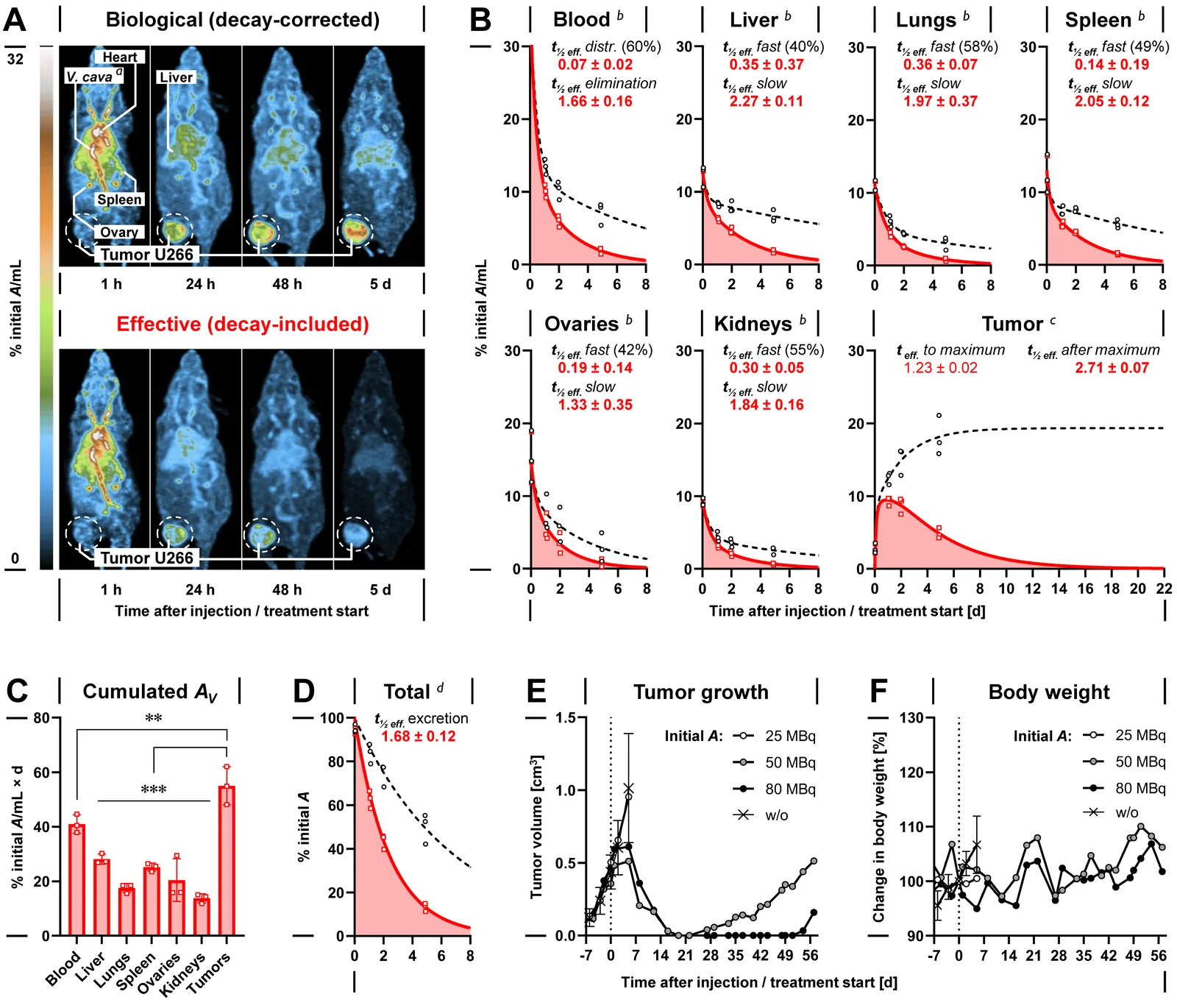
SPECT imaging, time-activity profiles, and a pilot study on treatment effects of **[^67^Cu]Cu-N_2_py_4_-MAB193** in mice bearing subcutaneous U266 myeloma xenografts; (A) SPECT images after treatment with 50 MBq; *^a^* signals from both *vena cava* and aorta; (B) Normalized time-activity curves extracted from SPECT images; (C) Normalized cumulated volume activity in tissues, (*n* = 3); (D) Total excretion excluding activity in tumors; dashed black lines: decay-corrected courses fitted with exponential regression models:*^ b^* ‘two-phase decay’, *^c^* ‘two-phase association’, *^d^* ‘one-phase decay’; continuous red lines: effective courses accounting for radionuclide decay; (E-F) Changes in tumor volume and body weight comparing untreated controls (w/o, *n* = 4) with radioimmunotherapy at individual initial activities (*n* = 1); dotted black line: treatment start, means ± standard deviation.

The suitability of the copper-67-labeled mAb **[^67^Cu]Cu-N_2_py_4_-MAB193** for SPECT image-based estimations of therapeutic doses was tested in three mice bearing subcutaneous U266 myeloma xenografts, and treatment effects were documented in the setting of a pilot study. Each of the three animals received a single treatment with an individual total activity of 25, 50, and 80 MBq, respectively. Quantitative SPECT imaging showed that both distribution and tumor accumulation of **[^67^Cu]Cu-N_2_py_4_-MAB193** were consistent with those of its copper-64-labeled counterpart (Figure 4 A and *Supporting information,* Table S 2 and Table S 5).

The pharmacokinetic profiles of **[^67^Cu]Cu-N_2_py_4_-MAB193** in the liver, lungs, spleen, ovaries, and kidneys showed similar shapes as in the blood (effective half-lives: distribution 1.68 h, elimination 39.8 h), indicating that the retention of the radiolabeled mAb in the vasculature is the primary contributor to the cumulated activity in normal tissues (Figure 4 B). Compared to normal tissues, BCMA-specific uptake of **[^67^Cu]Cu-N_2_py_4_-MAB193** in U266 tumors provided a significantly higher time-cumulated tissue activity (Figure 4 C). The initially administered activity was excreted with an effective half-life of 40.3 h (Figure 4 D). An increase in the total molar amount of **[^67^Cu]Cu-N_2_py_4_-MAB193** from 0.36 to 1.14 nmol had no effect on relative uptake and pharmacokinetic profiles.

Single treatment with **[^67^Cu]Cu-N_2_py_4_-MAB193** provided absorbed doses between 0.98 and 1.26 Gy/MBq in U266 tumors. By increasing the administered total activity from 25 to 80 MBq, the total absorbed doses in the tumors increased from 32.3 to 89.0 Gy. In two animals, total absorbed doses of >52 Gy were associated with a reduction in tumor volume and extension of progression-free survival, up from 0 d in the untreated control group to 29-55 d (Figure 4 E, Table 2). The relative changes in body weight following treatment with **[^67^Cu]Cu-N_2_py_4_-MAB193** remained below 10% (Figure 4 F).

The expected radiation exposure for humans undergoing radioimmunotherapy with **[^67^Cu]Cu-N_2_py_4_-MAB193** were predicted based on quantitative SPECT imaging of U266 myeloma-bearing mice (*Supporting information*, Table S 6 A). Organs that received the highest absorbed doses, ranging from 416 to 612 µGy/MBq, were spleen < ovaries < liver < heart wall. The predicted human effective doses were 162 µSv/MBq for women and 112 µSv/MBq for men, without taking tumor-specific accumulation into account. In comparison, the mean difference in dose estimates for **[^67^Cu]Cu-N_2_py_4_-MAB193** extrapolated from PET imaging using its corresponding copper-64-labeled counterpart was below 6% (Table S 6 B).

## Discussion

This preclinical study demonstrates the theranostic potential and practical applicability of the radionuclide pair copper-64 and copper-67 in BCMA-targeted immunotheranostics of MM, as indicated by the theranostic capabilities of experimental radiocopper-labeled bispidine-mAb conjugates directed against BCMA in models of human myeloma.

The starting point for this study was the pre-selection of three recombinant anti-BCMA mAbs **MAB193**, **Vicky-1**, and **E6D7B** as candidates for conversion into radiocopper-labeled bispidine-mAb conjugates, based on published results demonstrating their antigen-specific immunoreactivity 48-50, different antibody subtypes, and availability in sufficient quantities from commercial vendors.

With *K*_d_ values in the picomolar to nanomolar range (0.33-22 nM), the two BCMA-directed mAbs, **MAB193** and **Vicky-1**, meet the binding affinity requirements that warrant their conversion into radioimmunotheranostic agents. Their binding affinity is comparable to other biological vectors that have been recently converted into BCMA radiotracers 27, 30-32. Despite the reported BCMA-specific immunoreactivity of the third mAb candidate, **E6D7B,** which has been demonstrated in Western blot experiments and immunohistochemistry using formalin-fixed, paraffin-embedded tissue 50, 51, the antibody was excluded from further investigations due to insufficient binding to native human BCMA.

For labeling with radiocopper, **MAB193** and **Vicky-1** were modified using the bifunctional bispidine **N_2_py_4_-Bn-NCS** based on the following considerations: First, the NCS group enables straightforward lysine residue-directed bioconjugation of antibodies resulting in covalent thiourea bonds with desirable stability in buffers and human blood serum 52. Second, N_2_Py_4_ offers rapid and efficient complex formation with Cu^II^ under mild conditions (room temperature and neutral pH), particularly favorable for mAbs-based radioconjugates, as they prevent detrimental effects on immunoreactivity and pharmacokinetic properties, potentially resulting from harsh labeling conditions which are often required for radiometal complex formation with other chelating moieties 53. Third, the Cu-N_2_py_4_ complex exhibits high kinetic inertness which is especially important for radiocopper-labeled conjugates, due to the high abundance of copper-avid proteins *in vivo* 38, 54.

To characterize the pharmacokinetic properties of **[^64^Cu]Cu-N_2_py_4_-MAB193** and **[^64^Cu]Cu-N_2_py_4_-Vicky-1** in biological settings, U266 and L363 cells were used for generating complementary myeloma models that differ in their natural BCMA concentration. Additionally, A375 melanoma cells were included as a BCMA-negative control model to verify the antigen-specificity of tumor cell immunotargeting. The observation that U266 cells exhibit higher BCMA levels than L363 cells is consistent with previous reports 13, 55. The present results further demonstrate that the intrinsically different BCMA levels of U266 and L363 myeloma cells are maintained in the corresponding subcutaneous tumor xenografts in mice. In fact, subcutaneous tumor xenograft models were chosen because, at this early stage of radiotracer characterization, they allow for straightforward *in vivo* pharmacokinetic monitoring - with clearly identifiable tumor sites being a favorable prerequisite for extracting meaningful tumor uptake values from quantitative PET and SPECT images.

Comparative investigations that include both U266 and L363 tumors offer a valuable opportunity to test the BCMA-dependent tumor specificity of theranostic agents *in vivo* across different biological variants of the same tumor entity, i.e., requiring no genetic manipulation of *BCMA* gene expression or blockade experiments in presence of a BCMA-specific competitor. The higher non-specific uptake of **[^64^Cu]Cu-N_2_py_4_-MAB193** in the BCMA-negative A375 melanoma xenografts, compared to the L363 myeloma xenografts, exemplifies that characterizing the off-target uptake of radioimmunoconjugates in tumor models of a different entity can occasionally lead to false conclusions.

BCMA is known to be cleaved from the myeloma cell surface by γ-secretase, resulting in the release of sBCMA into the bloodstream 19. In myeloma-bearing mice, the effective reduction in sBCMA levels in the peripheral blood by systematic short-term treatment with GSI (maintained until 48 h after radiotracer injection) largely rules out any effects of circulating sBCMA on the blood kinetics of the radiocopper-labeled mAbs. This is further supported by the observation that, e.g., blood kinetics of **[^64^Cu]Cu-N_2_py_4_-MAB193** were the same in both GSI-treated myeloma-bearing mice and naïve A375 melanoma-bearing mice. However, it is known that GSI treatment with crenigacestat used in the present study affects several signaling pathways, including Notch signaling, and can alter tumor biology and microenvironment 56. For example, short-term GSI treatment in mice has been reported to increase cell surface levels of BCMA in MM.1R myeloma xenografts 57. Since this was not systematically investigated in the present study, BCMA-stimulatory and other biological effects of GSI treatment on myeloma xenografts cannot be ruled out. For this reason, GSI treatment was applied to all myeloma-bearing mice to ensure consistent experimental conditions across *in vivo* experiments.

Results from PET imaging in U266 myeloma-bearing mice and *ex vivo* analysis of tissue explants demonstrate that **[^64^Cu]Cu-N_2_py_4_-MAB193** is a suitable PET radiotracer for the specific detection of BCMA-positive myeloma xenografts, with encouragingly low background in normal tissues. On the other hand, insufficient tumor uptake and extensive liver background in the U266 myeloma xenograft model led to the exclusion of **[^64^Cu]Cu-N_2_py_4_-Vicky-1** from further investigations. It can be assumed that the almost instantaneous trapping of **[^64^Cu]Cu-N_2_py_4-_Vicky-1** in the liver, which results in rapid clearance from the blood, is the main reason for its low tumor uptake. However, the exact mechanisms underlying the vastly different liver accumulation of the two radiocopper-labeled bispidine-mAb conjugates remain elusive at this stage.

Taking into account both the concentration of a tissue-specific molecular target and the binding affinity of a target-specific vector, a *B*_max_/*K*_d_ ratio ≥ 10 has been proposed to identify target/vector combinations that promise sufficient tissue contrast in molecular *in vivo* imaging 58. The binding constants measured on tumor cryosections demonstrate that the BCMA-specific recognition of U266 myeloma xenografts by **[^64^Cu]Cu-N_2_py_4_-MAB193** exceeds this benchmark, providing a *B*_max_/*K*_d_ ratio of approximately 40.

**[^64^Cu]Cu-N_2_py_4_-MAB193** showed no binding to BCMA-negative A375 melanoma cryosections *in vitro*, indicating that its non-specific tumor uptake *in vivo* is likely not driven by cross reactivity with non-BCMA antigens or other specific molecular interactions. This is further supported by results from flow cytometry demonstrating no relevant expression of potentially antibody-capturing Fc receptors in A375 melanoma cells, nor in U266 and L363 myeloma cells. Therefore, non-specific uptake of **[^64^Cu]Cu-N_2_py_4_-MAB193** in both melanoma and myeloma xenografts *in vivo* is most likely attributable to target-independent mechanisms, such as ‘enhanced permeability and retention’ (EPR). Especially subcutaneous tumor models are prone to EPR-driven effects 59. This suggests, for example, that EPR-driven effects may account, for 26-33% of the total uptake in U266 myeloma xenografts.

As a PET radiotracer, **[^64^Cu]Cu-N_2_py_4_-MAB193** showed no accumulation in most normal mouse tissues. However, a moderate uptake in the liver followed by slower clearance from the hepatic tissues compared to the blood suggests metabolic processing in the liver. Since its metabolic profile was not investigated in this study, no definitive conclusions can be drawn at this stage regarding the potential implications of radiometabolites for accumulation in the liver. However, referring to previous studies, it seems unlikely that a release of [^64^Cu]Cu^2+^ has any major role in liver accumulation, since the intact Cu-N_2_py_4_ complex has shown high resistance to demetalation in blood serum and to transchelation by physiological competing ligands present in the murine liver 38, 60. Furthermore, there is no evidence from our previous investigations that might raise serious concerns about the stability of the thiourea bond in chelator-mAb conjugates 52.

The tumor uptake of **[^64^Cu]Cu-N_2_py_4_-MAB193**, detected in PET images 24 h after injection in U266 myeloma-bearing mice (SUVmean 2.74; equiv. to ~ 9.13% initial A/mL), is within the range reported for other radiolabeled mAbs targeting BCMA in subcutaneous myeloma xenograft models, for example, **[^89^Zr]Zr-DFO-BCMAh230430** in MM.1S tumors (~5% initial A/mL) 30 and **[^89^Zr]Zr-DFO-PFBH0L0** in H929 tumors (~10% initial A/mL) 31. In PET imaging based on copper-64-based radiotracers, the further accumulation of mAbs in tumors – as also observed for **[^64^Cu]Cu-N_2_py_4_-MAB193** reaching tumor-to-muscle ratios up to 14.9 in U266 myeloma-bearing mice after 48 h - may be of limited relevance in current clinical settings due to the progressive decay of copper-64 and the resulting decrease in signal intensity. On the other hand, this limitation can be overcome by use of modern long axial field of view (LAFOV) PET scanners, which offer higher sensitivity compared to conventional PET scanners 61.

As an advantage over to the zirconium-89-labeld mAbs, both of which exhibit high liver background as well as a significant release and bone accumulation of [^89^Zr]Zr^4+^ *in vivo* 30, 31, the lower normal tissue background of **[^64^Cu]Cu-N_2_py_4_-MAB193** and its non-bone-avid radiolabel suggests that BCMA-targeted immunoPET using a copper-64-labeled bispidine-mAb conjugate holds promise for improved lesion detection, particularly in bones. This requires further examination in future studies. Furthermore, the predicted human effective dose for immunoPET using **[^64^Cu]Cu-N_2_py_4_-MAB193** (25 and 35 µSv/MBq in adult women and men, respectively) corresponds to only 4-10% of the radiation exposure predicted for the use of zirconium-89-labeled mAbs (344 and 680 µSv/MBq) 30, 31.

Besides mAb-based approaches, other BCMA-specific vector molecules have been converted into gallium-68- and fluorine-18-containing PET radiotracers, including peptides 29 and sdAbs 27, 32, 33. However, using these PET radiotracers for prospective therapeutic dose planning is challenging because their therapeutic counterparts are usually not chemically identical and contain radionuclides with a considerably longer physical half-life, as exemplified by the theranostic pair of sdAbs **[^18^F]FPy-BCMA-Nb** and **[^131^I]I-BCMA-Nb** 32. Other PET radiotracers, such as the peptide-based **[^68^Ga]Ga-DOTA-BP1** 29 and sdAb-based **[^68^Ga]Ga-NOTA-MMBC** derivatives 27, have shown uptake values below 0.6% initial A/mL in myeloma xenografts and fast washout from the tissue, presumably limiting the anti-tumor efficacy of their radiotherapeutic counterparts.

Compared to the existing BCMA-targeted peptide- and sdAb-based PET radiotracers containing short-lived radionuclides 27, 29, 32, copper-64-labeled bispidine-mAb conjugates unfold their advantage in light of existing chemically identical, copper-67-labeled counterparts, for which copper-64 provides a sufficiently long imaging window to enable prospective therapeutic dose planning. This is supported by the fact that the dose estimates derived directly from treatment of mice with **[^67^Cu]Cu-N_2_py_4_-MAB193** are consistent with those extrapolated from PET imaging using its copper-64-labeled diagnostic counterpart. Furthermore, lower renal accumulation of **[^64^Cu/^67^Cu]Cu-N_2_py_4_-MAB193** points towards lower radiation exposure to kidneys associated with BCMA-targeted immunotheranostics based on radiocopper-labeled bispidine-mAb conjugates. On the other hand, their longer blood retention and slower tumor accumulation point towards higher radiation exposure to blood and bone marrow cells, as well as slower build-up of the desired tumor-to-background contrast in PET imaging.

As an advantage over copper-64, the longer physical half-life of copper-67 allowed for monitoring **[^67^Cu]Cu-N_2_py_4_-MAB193** in U266 tumor-bearing mice for 5 d using quantitative small animal SPECT imaging. Its extended traceability increased the accuracy of the extrapolated time-activity profiles for normal tissues and tumors, and thus dose estimates. This suggests that BCMA-targeted therapies using copper-67-labeled bispidine-mAb conjugates offer sufficiently long imaging windows to allow for precise SPECT-based dose monitoring. Given the high abundance of γ photons emitted during nuclear conversion of copper-67 5, 6, a low-activity use of copper-67-labeled bispidine-mAb conjugates may also be considered for prospective SPECT-based dose planning with a favorable imaging window.

In mice, radiolabeled mAbs of the IgG type directed against tumor-specific molecular targets typically show biological half-lives in the blood between 4 and 10 d 62, 63, and reach their peak concentrations in solid tumors between 1 and 4 d, occasionally within up to 7 d after injection 64-67. **[^67^Cu]Cu-N_2_py_4_-MAB193** is well within these ranges, reaching its highest pharmacological concentration in U266 myeloma xenografts between 2 and 5 d after injection. However, less than 58% of the initially possible radiation dose per pharmacological amount of **[^67^Cu]Cu-N_2_py_4_-MAB193** remain to be delivered to the tumor tissue from 2 d onwards (within another 2 weeks), due to the already advanced decay of copper-67 at the time point when the antibody approaches its highest pharmacologic concentration in U266 tumors.

Whereas the accumulation of IgG type antibodies in solid tumors frequently follows similar time courses in both mice 62-66 and humans 64, 68-71, their elimination from the blood is usually slower in humans, with biological half-lives between 20 and 24 d 72. Consequently, it can be assumed that the ratio of radiation exposure between tumor and blood will be lower in a clinical setting of BCMA-targeted therapy using copper-67-labeled bispidine-mAb conjugates compared to the preclinical setting.

Despite the relatively slow accumulation of **[^67^Cu]Cu-N_2_py_4_-MAB193** in U266 myeloma xenografts, single-dose treatment provided absorbed tissue doses between 0.96 and 1.27 Gy/MBq in the tumors. This is comparable to the absorbed tumor doses delivered by clinically approved β‾ particle-emitting radiopharmaceuticals addressing other critical cancer targets in preclinical settings, for example by **[^177^Lu]Lu-PSMA-617** in PSMA-positive prostate cancer models (0.22-0.68 Gy/MBq) 73, and by** [^177^Lu]Lu-DOTA-TATE** in SSTR2-positive neuroendocrine tumor models (0.27-0.82 Gy/MBq) 5, 74-76.

Single-dose treatments with **[^67^Cu]Cu-N_2_py_4_-MAB193** that delivered total absorbed doses >52 Gy in U266 myeloma xenografts reduced the tumor mass and prolonged the progression-free survival of mice. This suggests that BCMA-targeted radioimmunotherapy using copper-67-labeled bispidine-mAb conjugates can be effective against MM, although no conclusions on dose-response relationships can be drawn from the pilot study at this stage. Nevertheless, the sensitivity of U266 myeloma xenografts to treatment with **[^67^Cu]Cu-N_2_py_4_-MAB193** is in line with other reports demonstrating therapeutic efficacy of β‾ particle-emitting immunoconjugates targeting BCMA in myeloma xenograft models, for example sdAb- and mAb-based agents labeled with iodine-131 and lutetium-177, respectively 30, 32.

Short-term GSI treatment has been reported to exert no growth-reducing effects on MM.1R myeloma xenografts in mice 57. It can therefore be assumed that this treatment regimen has no effect on U266 tumor growth in the present study either. Furthermore, it has been reported that an unconjugated anti-BCMA mAb, **J6M0** (*K*_d_ = 1 nM), slowed the growth of subcutaneous H929 myeloma xenografts in mice but did not reduce tumor mass, even when up to four doses of 4 mg/kg (~0.8 nmol/30-g-animal) were administered twice weekly (total cumulated dose of ~3 nmol/animal) 77. Therefore, it can be assumed that the efficient reduction of the U266 tumor mass following treatment with **[^67^Cu]Cu-N_2_py_4_-MAB193** at single molar doses of 0.72 and 1.12 nmol/animal, is predominantly attributable to the radiotoxic effects of copper-67. However, the anti-tumor effects of the unconjugated **MAB193** were not investigated in the pilot study herein.

The observation that tumors regressed while the animals maintained their body weight and remained in good general health, even in response to treatment with **[^67^Cu]Cu-N_2_py_4_-MAB193** at an initial activity dose of up to 80 MBq, provides a solid foundation for the future therapeutic evaluation of BCMA-targeted therapies using copper-67-labeled radioimmunoconjugates; however, it does not yet allow for definitive conclusions regarding safety of this approach.

The effective doses predicted for BCMA-targeted radioimmunotherapy in humans using **[^67^Cu]Cu-N_2_py_4_-MAB193** (less than 161µSv/MBq) are lower compared to other BCMA-directed mAbs, such as **[^89^Zr]Zr-DFO-BCMAh230430** (344 µSv/MBq) 30 and **[^89^Zr]Zr-DFO-PFBH0L0** (680 µSv/MBq) 78, but align more closely with the effective doses predicted for mAbs that target other critical cancer antigens and have yet been included in clinical trials, for example, anti-PSMA **[^177^Lu]Lu-DOTA-rosopatamab** (210 µSv/MBq) 79 and anti-CD20 **[^177^Lu]Lu-DOTA-rituximab** (220 µSv/MBq) 80. Of note, the effective dose predicted for the copper-67-labeled anti-HER2 **[^67^Cu]Cu-NOTA-trastuzumab** (17 µSv/MBq) 9 may have underestimated the potential radiation exposure in humans, although its activity concentrations in mouse tissues are comparable to those of **[^67^Cu]Cu-N_2_py_4_-MAB193** in the preclinical setting.

The predicted radiation exposure in humans from treatment with **[^67^Cu]Cu-N_2_py_4_-MAB193** is more than 2.3-fold higher compared to clinically approved peptide-based radiopharmaceuticals such as **[^177^Lu]Lu-PSMA-617** (50 µSv/MBq) 81 and **[^177^Lu]Lu-DOTA-TATE** (70 µSv/MBq) 82, which are used to treat prostate cancer and neuroendocrine tumors, respectively. This suggests that the further development of BCMA-targeted radioimmunotherapies based on copper-67 requires immunoconjugates offering both faster uptake in tumors and faster elimination from the blood.

This preclinical study has limitations. Besides their usefulness for early-stage radiopharmaceutical characterization, subcutaneous myeloma models do not reflect the typical clinical manifestation of MM. Clinically, MM is a disseminated systemic malignancy characterized by diffuse bone marrow infiltration, where vascular permeability, antigen accessibility, and microenvironmental interactions may differ substantially from the subcutaneous tissue. Subcutaneous models are prone to EPR-driven effects, which may artificially inflate tumor accumulation of large molecules such as mAbs. Therefore, tumor uptake and pharmacokinetics of radiolabeled mAbs, as well as therapeutic responses may differ between subcutaneous and disseminated bone marrow disease. However, in one recent study, a fluorine-18-labeled BCMA-specific sdAb has shown comparable uptake in both subcutaneous and disseminated myeloma xenografts 32. On the other hand, alpha-radioimmunotherapy using an astatine-211-labeled mAb directed against CD38 has been reported to be less effective in subcutaneous compared to disseminated models of MM 83. Furthermore, radiation doses predicted based on the distribution of BCMA-targeted radioimmunoconjugates in mice bearing subcutaneous myeloma xenografts may underestimate bone marrow exposure in humans potentially resulting from uptake in diffuse and intermedullary lesions.

Due to the species selectivity of **[^64^Cu/^67^Cu]Cu-N_2_py_4_-MAB193** for human BCMA, whose occurrence in myeloma xenograft mice is restricted to the human tumor tissue (as well as to tumor-derived human sBCMA in peripheral blood), the potential contributions of physiologic BCMA to the uptake in normal mouse tissues remained undetectable in the present study.

It should also be noted that **[^64^Cu/^67^Cu]Cu-N_2_py_4_-MAB193** itself does not meet the molecular requirements for direct medical use. Efforts towards clinical translation require molecular engineering of its Fc region (rat IgG_2A_) to generate a humanized chimeric mAb which is compatible with the human immune system and bypasses human Fcγ receptor-mediated uptake in normal tissues.

To more precisely tailor the pharmacokinetic properties of BCMA-targeted immunotherapeutic agents for their use with radiocopper - particularly with regard to faster blood kinetics - various strategies are known to be effective and may therefore be considered in future developments, including pretargeting 84, the use of antibody-nanoparticle conjugates 28, or antibody fragments with smaller molecular weights such as sdAbs 27, 32, 33, 40, minibodies and diabodies 85, or Fab fragments 86.

## Conclusion

The fundamental efficacy of copper-64 and copper-67 in BCMA-targeted immunotheranostics of MM promises both precise PET-based dose planning and radioimmunotherapy in combination with highly sensitive SPECT-based dose monitoring, as demonstrated by the theranostic capabilities of the chemically identical bispidine-mAb conjugates **[^64^Cu]Cu-N_2_py_4_-MAB193** and **[^67^Cu]Cu-N_2_py_4_-MAB193** in tumor-bearing mice. The results provide a strong incentive for incorporating the *CopperNostics* approach into the further development of BCMA-targeted radioimmunotheranostic agents, including precise tailoring of their pharmacokinetic properties to the physical half-life of copper-67.

## Supplementary Material

Supplementary methodological details and additional results (images, tables, graphs).

## Figures and Tables

**Table 1:** Binding affinities (*K*_d_ values, given in nM) of pristine anti-BCMA mAbs towards recombinant and native cellular human BCMA; *K*_d_ values given as (a) means ± standard error resulting from SPR interaction analysis (*n* = 2, each analyzed in triplicate) or (b) means ± standard deviation resulting from flow cytometry measurements (*n* = 3); (n.d.) not determined

Sample	MAB193	Vicky-1	E6D7B
recombinant human BCMA^ a^	0.33 ± 0.02	0.38 ± 0.01	no binding
recombinant murine BCMA^ a^	no binding	no binding	no binding
human BCMA-positive U266 cells^ b^	21.7 ± 11.1	11.1 ± 6.2	n.d.
human BCMA-positive L363 cells^ b^	7.80 ± 3.70	6.3 ± 0.9	n.d.

**Table 2:** Absorbed doses in U266 myeloma xenografts and survival of tumor-bearing mice after treatment with **[^67^Cu]Cu-N_2_py_4_-MAB193**; Conditions at start of observation/treatment: (BW) body weight, (*V*_Start_*,*_Tumor_) tumor volume, (IA) initial activity dose, (ID_ molar_) initial molar dose; (AUC_ Tumor_) area under normalized time-activity curves also referred to as normalized cumulated volume activity in tumors; (*D_T_*,_ Tumor_) absorbed tissue dose in tumors calculated using the ‘Sphere’ model implemented in Olinda 2.2.3, (PFS) progression-free survival defined as < 50 mm^3^ increase in tumor volume compared to minimum; (w/o, *n* = 4) untreated controls; means ± standard deviation

Animal	BW_Start_	*V*_Start, Tumor_	Treatment	IA	ID _molar_	AUC _Tumor_	*D_T,_*_Tumor_ per MBq	*D_T,_*_Tumor_	PFS
#	[g]	[cm^3^]		[MBq]	[nmol]	[% IA/mL×d]	[Gy/MBq]	[Gy]	[d]
1-4	29.6 ± 2.99	0.44 ± 0.12	w/o	-	-	-	-	-	0
5	29.2	0.50	[^67^Cu]Cu-N_2_py_4_-MAB193	25	0.36	62.1	1.26	32.3	0
6	30.4	0.36	[^67^Cu]Cu-N_2_py_4_-MAB193	50	0.71	48.1	0.98	52.0	29
7	31.4	0.47	[^67^Cu]Cu-N_2_py_4_-MAB193	80	1.14	54.9	1.12	89.0	55

## Data Availability

Beyond the supplementary material, additional raw data can be provided upon request; please contact the corresponding author.

## Abbreviations

*A*_m_: molar activity
BCMA: B cell maturation antigen
CT: computed tomography
EDTA: ethylenediaminetetraacetic acid
GSI: gamma secretase inhibitor
IgG: immunoglobulin G
ITC: isotype control
mAb: monoclonal antibody
MALDI-TOF: matrix-assisted laser desorption/ionization time-of-flight mass spectrometry
MM: multiple myeloma
PET: positron emission tomography
sBCMA: soluble B cell maturation antigen
sdAb: single-domain antibody
SPECT: single-photon emission computed tomography
SPR: surface plasmon resonance
SUVmean: region-averaged standardized uptake value
TLC: thin layer chromatography
