# Supplementary material for: Radiocopper in BCMA-targeted immunotheranostics of myeloma
Source: Theranostics. 2026 Jul 29;16(15):8461–77. doi: 10.7150/thno.134397 (PMC13549754; doi:10.7150/thno.134397)
Supplement: Supplementary file 1 [file thnov16p8461s1.pdf]

- **Supporting Information –**

Radiocopper in BCMA-targeted immunotheranostics of myeloma

Martin Ullrich[1#], Kristof Zarschler[2], Manja Kubeil[1,3], Markus Laube[1], Jens Pietzsch[1,4*], and Birgit Belter[1*]

[1]Helmholtz-Zentrum Dresden-Rossendorf, Institute of Radiopharmaceutical Cancer Research, Department of Radiopharmaceutical and Chemical Biology, Bautzner Landstrasse 400, D-01328 Dresden, Germany
[2]Helmholtz-Zentrum Dresden-Rossendorf, Institute of Radiopharmaceutical Cancer Research, Department of Medicinal Radiochemistry, , Bautzner Landstrasse 400, D-01328 Dresden, Germany
[3]Helmholtz-Zentrum Dresden-Rossendorf, Institute of Resource Ecology, Department of Radiation Research on Biological Systems, , Bautzner Landstrasse 400, D-01328 Dresden, Germany
[4]Technische Universität Dresden, School of Science, Faculty of Chemistry and Food Chemistry, D-01062 Dresden, Germany

*J.P. and B.B. share senior authorship

Abbreviated Title: Radiocopper-labeled BCMA antibodies

Keywords: multiple myeloma, B cell maturation antigen, small animal PET, small animal SPECT, xenograft model, copper-64, copper-67, bispidine chelators

[#]Corresponding author and person to whom reprint requests should be addressed:

Martin Ullrich, Ph.D.

Helmholtz-Zentrum Dresden-Rossendorf

Institute of Radiopharmaceutical Cancer Research

Department of Radiopharmaceutical and Chemical Biology

Bautzner Landstraße 400, 01328 Dresden, Germany

Phone: +49-351-260 4046, Fax: +49-351-260 12622

E-mail: m.ullrich@hzdr.de

Table of Contents

I. Additional materials and methods 3

Surface plasmon resonance: Binding of pristine mAbs to recombinant BCMA 3

Mass spectrometry: Quality control of bispidine-mAb conjugates 4

Thin layer chromatography: Quality control of radiolabeled bispidine-mAb conjugates 4

Gel electrophoresis: Quality control of radiolabeled bispidine-mAb conjugates 4

Flow cytometry: Cell line-specific BCMA concentrations and binding affinities of different pristine mAbs 4

Quantitative analysis of PET and SPECT images: Extraction of tissue-specific uptake values 5

Biodistribution measured ex vivo: Uptake of mAbs in tissue explants 6

Ex vivo radioluminography of tissue cyrosections: Uptake of mAbs in tumors and reference tissues 6

Flow cytometry: BCMA target specificity test for MAB193 6

In vitro binding on tissue cryosections: BCMA target specificity test for **[^{64}Cu]Cu-N$_2$py$_4$-MAB193** 7

Flow cytometry: Fc receptor levels of myeloma and melanoma cells 7

II. Binding of mAbs to recombinant BCMA 7

Binding kinetics and binding constants: Human BCMA 7

Species-selectivity: Human versus murine BCMA 10

III. Bioconjugation of mAbs with a bispidine chelator 11

Chemical properties of bispidine-mAb conjugates 11

IV. Cellular binding of mAbs and bispidine-mAb conjugates 13

Binding constants for mAbs and bispidine-mAb conjugates towards BCMA-positive myeloma cells 13

V. Radiolabeling of bispidine-mAb conjugates 15

Radiochemical properties of copper-64 and copper-67-labeled N$_2$py$_4$-mAb conjugates 15

VI. Distribution of copper-64-labeled anti-BCMA mAbs *in vivo* 17

[^{64}Cu]Cu-N$_2$py$_4$-Vicky-1: PET images and image-derived uptake values in U266 xenograft mice 17

[^{64}Cu]Cu-N$_2$py$_4$-MAB193 versus **[^{64}Cu]Cu-N$_2$py$_4$-Vicky-1**: Ex-vivo uptake values in tissue explants from U266 xenograft mice 17

[^{64}Cu]Cu-N$_2$py$_4$-MAB193 versus **[^{64}Cu]Cu-N$_2$py$_4$-Vicky-1**: Radioluminographic analysis of uptake in U266 tumors 18

[^{64}Cu]Cu-N$_2$py$_4$-MAB193: PET image-derived uptake values in tumor-bearing mice 19

[^{64}Cu]Cu-N$_2$py$_4$-IgG$_{2A}$-ITC (MAB193 isotype control): PET image-derived uptake values in tumor-bearing mice 20

[^{64}Cu]Cu-N$_2$py$_4$-MAB193: Predicted human absorbed and effective doses 21

VII. BCMA target specificity of [^{64}Cu]Cu-N$_2$py$_4$-MAB193 22

MAB193 and **[^{64}Cu]Cu-N$_2$py$_4$-MAB193**: BCMA-specificity of binding to cells and tissue cryosections 22

VIII. Distribution of [^{67}Cu]Cu-N$_2$py$_4$-MAB193 in vivo 23

[^{67}Cu]Cu-N$_2$py$_4$-MAB193: SPECT image-derived uptake values in U266 xenograft mice 23

[^{67}Cu]Cu-N$_2$py$_4$-MAB193: Predicted human equivalent and effective doses 24

IX. Fc receptor levels in myeloma cells 25

Fc receptor levels of U266, L363 myeloma cells and A375 melanoma cells 25

I. Additional materials and methods

Surface plasmon resonance: Binding of pristine mAbs to recombinant BCMA

Surface plasmon resonance (SPR) interaction analysis was carried out on a Biacore T200 (GE Healthcare, Chicago, IL, USA) at 25 °C using CM5 sensor chips and HBS-P+ as running buffer (both Cytiva) at a flow rate of 30 µL/min unless otherwise specified, and a data collection rate of 10 Hz. All flow cells (FC) were normalized and functionalized using the Amine Coupling and His Capture Kits (both Cytiva). Functionalization involved surface activation (*1*-Ethyl-*3*-(*3*-dimethylaminopropyl)carbodiimide)/*N*-hydroxysuccinimide), anti-His antibody coupling (35 µg/mL in 10 mM acetate buffer pH 4.5, exposure time 420 s, flow rate 5 µL/min), and surface blocking (1 M ethanolamine pH 8.5). Functionalization resulted in 6878 RU on FC(active) and 6554 RU on FC(reference).

Binding analyses were performed by a capturing approach which involves binding of His-tagged human BCMA to the anti-HIS antibody as ligand on FC(active) followed by binding analysis of mAbs to the surface bound BCMA by a single-cycle-kinetic on both FC. To equilibrate the flow system and bound anti-HIS mAbs on the chip surface before a new experimental set, three start-up cycles were performed, each involving the capturing of His-tagged human BCMA (on FC(active) only, 0.25–0.5 µg/mL, 60 s, flow rate 10 µL/min) and a blank injection (HBS-P+, 120 s). Regeneration was performed using 10 mM glycine (pH 1.5, 30 s) followed by a stabilization time of 300 s. For binding analysis, His-tagged human BCMA was captured on FC(active) only (0.25–0.5 µg/mL, 12 s, flow rate 10 µL/min) followed by a stabilization time of 450 s. Then, single-cycle kinetics were performed using both, FC(reference) followed by FC(active), by sequential exposure to a series of analyte samples containing five mAb concentrations increasing in 5-fold steps (0.032−20 nM, 240 s contact time for each concentration, flow rate 10 µL/min) and following dissociation for 900 s. Regeneration was performed as described above. Blanks (HBS-P+ without analyte) were measured accordingly. Time-resolved responses were recorded as resonance units (RU) and displayed as sensorgrams. Double-referenced sensorgrams were obtained by reference-subtraction (FC(active)−FC(reference)) and blank-correction (RUmean of blanks). Data was analyzed using the Biacore Evaluation 3.0 software. Binding constants were calculated from curve fitting using a 1:1 binding model. Two independent experiments were performed in triplicate and analyzed separately.

For investigating the species-selectivity of anti-human BCMA mAbs, binding analyses were performed using SPR as described above with some modifications: In brief, binding of mAbs to His-tagged human BCMA and His-tagged murine BCMA (Cat. No. BCA-M52H3, Acro Biosystems, Basel, Switzerland) was

directly compared. The antibody concentration range in analyte samples was 0.08–50 nM. **Anti-mouse BCMA-IgG** (Cat. No. MAB5931, Bio-Techne) was included as a positive control for capturing murine BCMA.

Mass spectrometry: Quality control of bispidine-mAb conjugates

Matrix-assisted laser desorption ionization time-of-flight (MALDI-TOF) mass spectrometry was performed using an autoflex maX TOF/TOF MALDI-MS (Bruker Daltonics, Bremen, Germany) as described recently [87]. Spectra were acquired in positive linear mode in combination with flexControl version 3.4 (Bruker Daltonics) and analyzed with flexAnalysis version 3.4 (Bruker Daltonics). An aqueous solution containing 5 mg/mL (w/v) fatty acids and globulin free human serum albumin (Merck) was used for calibration.

Thin layer chromatography: Quality control of radiolabeled bispidine-mAb conjugates

Radio-thin layer chromatography (radio-TLC) was performed using (i) neutral alumina plates coated with silica gel (Macherey-Nagel, Düren, Germany) as stationary phase and acetonitrile/H_2O (70:30, v/v) as mobile phase as well as (ii) glass microfiber chromatography paper (Merck) impregnated with silica gel (iTLC-SG) and 50 mM EDTA (pH 5.5) as mobile phase. The air-dried TLC strips were scanned with an Amersham Typhoon 5 Scanner (Cytiva, Marlborough, MA, USA) and the images were analyzed using Image-Quant TL version 8.1 (GE Healthcare).

Gel electrophoresis: Quality control of radiolabeled bispidine-mAb conjugates

Denaturing sodium dodecyl sulfate-polyacrylamide gel electrophoresis (SDS-PAGE) was carried out according to a standard protocol [88]. For each gel, PageRuler Plus Prestained Protein Ladder (Thermo Scientific) was used as size standard. After electrophoresis, protein bands were stained with PageBlue protein staining solution (Thermo Scientific) according to the manufacturer's instructions.

Flow cytometry: Cell line-specific BCMA concentrations and binding affinities of different pristine mAbs

Single-cell suspensions containing 1–2 × 10^6 U266, L363, and A375 cells per sample were analyzed using flow cytometry. Cell suspensions were incubated with the primary antibodies (5 µg/mL) at 4°C for 30 min, followed by secondary antibody at 4°C for 30 min in the dark. Cells incubated with secondary antibody only were used as negative control. After staining, the fluorescence intensity of cells was measured immediately using an Attune NxT Acoustic Focusing Cytometer (Fisher Scientific GmbH, Schwerte, Germany). Data was analyzed using Attune NxT Software.

The following primary anti-human BCMA mAbs were used: **MAB193**, **Vicky-1**, **E6D7B**, and **IgG$_{2A}$-ITC** (rat mAb isotype control for MAB193, Cat. No. MAB006, Bio-Techne). The following secondary antibodies were used: anti-Rat-Alexa Fluor™ 647 (Chicken IgY, Cat. No. A21472, Thermo Scientific), anti-Rat-Alexa Fluor™ 488 (Chicken IgY, Cat. No. A21470, Thermo Scientific), anti-Rabbit-Alexa Fluor™ 647 (goat IgG, Cat. No. A21244, Thermo Scientific), anti-Mouse-Alexa Fluor™ 647 (Chicken IgY, Cat. No. A21463, Thermo Scientific).

Quantitative analysis of PET and SPECT images: Extraction of tissue-specific uptake values

PET and SPECT images were post-processed and analyzed using Rover version 3.0.77h (ABX GmbH, Radeberg, Germany) and displayed as maximum intensity projections with common scaling over indicated time points. For extraction of region-averaged standardized uptake values (SUVmean), regions of interest (ROIs) were generated within spherical preselection masks including voxels above tissue-specific intensity thresholds (% of image's maximum voxel intensity) as follows: heart (> 60%, 10 mm^3 of blood content), liver (> 0%, 30 mm^3 of a central lobe), lung (> 0%, 30 mm^3 of the left lobe, spleen (> 50%, 30 mm^3 of a central region), ovaries (> 50%), kidneys (> 50%, 170 mm^3), muscle (0%, 30 mm^3 of triceps brachii), tumor (> 30%), urinary bladder (> 10%), and total body (> 1%, urinary bladder excluded). ROI positions are exemplified in Figure S 1.

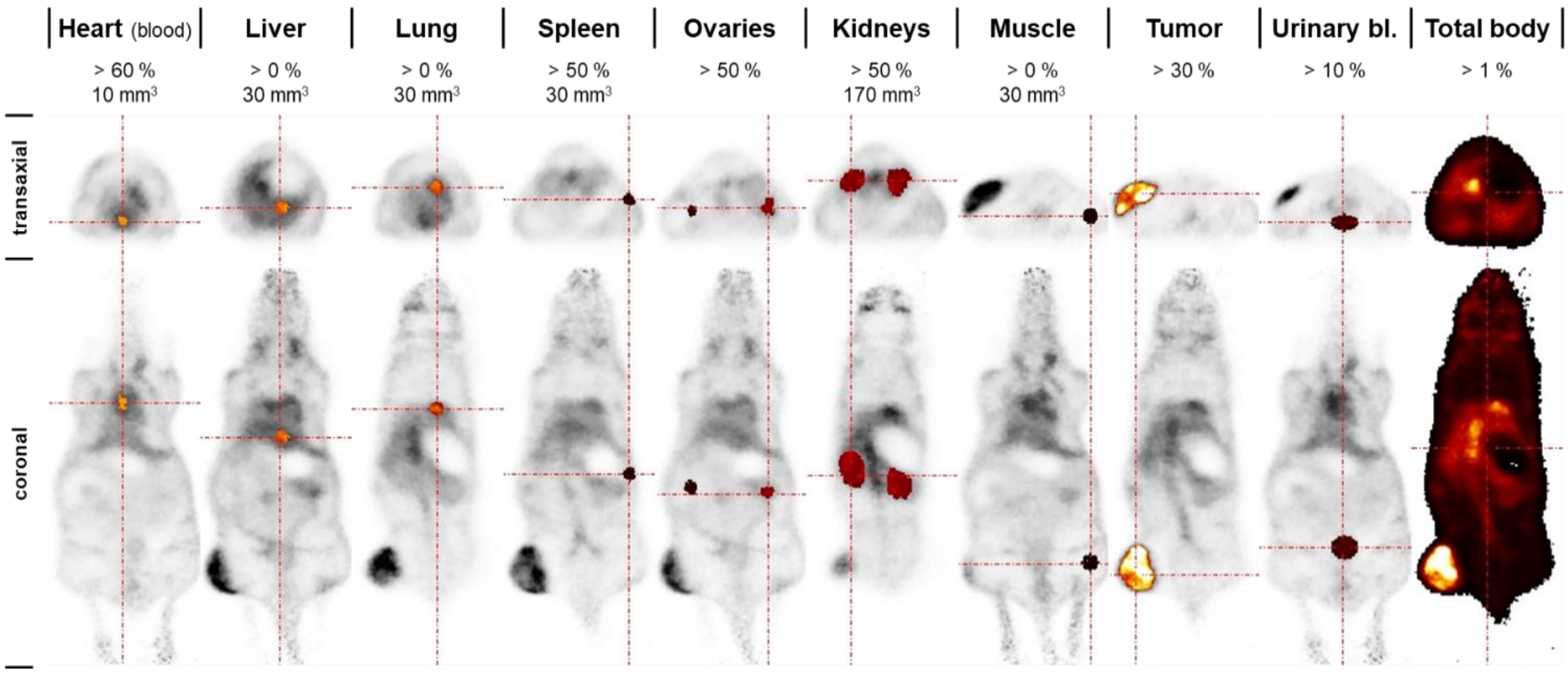

Figure S 1: Regions of interest (ROIs) generated in Rover for extraction of tissue-specific uptake values from PET images; exemplary data showing position (red crosshairs) and shape (black-to-yellow overlays) of the ROIs in orthogonal PET image slices of a U266 tumor-bearing mouse 24 h after injection of **[^{64}Cu]Cu-N$_2$py$_4$-MAB193**; percentages refer to tissue-specific intensity thresholds (% of image's maximum voxel intensity) used for including voxels into the ROI; volumes of ROIs given in mm^3; orientation: right | left

Biodistribution measured ex vivo: Uptake of mAbs in tissue explants

U266 tumor-bearing mice received a single intravenous injection of **[^{64}Cu]Cu-N$_2$py$_4$-MAB193** (0.71 ± 0.03 MBq, pharmacologically equivalent to 7.40 ± 0.27 pmol) or **[^{64}Cu]Cu-N$_2$py$_4$-Vicky-1** (0.17 ± 0.01 MBq, pharmacologically equivalent to 3.60 ± 0.10 pmol), each delivered in 0.2 mL isotonic NaCl (aq.) into a lateral tail vein. Mice were sacrificed by cervical dislocation at 24 h after injection and tissues of interest were excised. Tissue explants were weighed, and tissue-specific activity was measured using the Wizard™3'' gamma counter (PerkinElmer, Waltham, MA, USA). With each sample series, a series of activity standards was measured to convert counts/sample into Bq/sample. Tissue-specific uptake of copper-64-labeled bispidine-mAb conjugates was calculated and reported as SUV or percent of the initially administered dose.

Ex vivo radioluminography of tissue cyrosections: Uptake of mAbs in tumors and reference tissues

Copper-64-labeled bispidine-mAb conjugates were intravenously injected in U266 tumor-bearing mice as described in the methods section "PET imaging". After 24 h, animals were sacrificed, tumors and muscles were excised, quickly frozen in 2-methylbutane at −40° C, and stored in liquid nitrogen. Tissue cryosections (10 µm) were prepared and mounted on glass slides. A BAS-SR phosphoimaging plate was exposed to the tissue sections for 45 min in the dark and measured using an Amersham Typhoon™ FLA 9500 (Cytiva). Images were further processed and analyzed using AIDA 5.1 (Elysia-raytest). Tissue-specific regions-of-interest were drawn, region-averaged photostimulated luminescence (PSL) intensities were measured, and tumor-to-muscle ratios were calculated.

Flow cytometry: BCMA target specificity test for MAB193

Single-cell suspensions containing 1×10^6 to 2×10^6 cells per sample were analyzed using flow cytometry. **MAB193** at a fixed concentration (66 pM) was preincubated with the competitor anti-human BCMA sdAb **269A37948** at increasing concentrations (0.13–66 nM, corresponding to a 2–1000-fold molar excess) for 20 min at room temperature. U266 cells were incubated with the **MAB193**/sdAb solutions for 30 min at 4 °C, followed by incubation with a fluorophore-labeled secondary antibody (goat anti-rat Alexa488, Cat. No. A11006, Thermo Scientific) at 4 °C for 30 min in the dark. Cells incubated with secondary antibody only served as negative control. The relative fluorescence intensity (RFU) of cells was measured using the Attune NxT Acoustic Focusing Cytometer (Thermo Scientific). Data was analyzed using the Attune NxT Software.

In vitro binding on tissue cryosections: BCMA target specificity test for ***[^{64}Cu]Cu-N$_2$py$_4$-MAB193***

Tumor-bearing animals were sacrificed by cervical dislocation under anesthetic. Tumor as well as muscle from the contralateral site and a piece of liver were excised, quickly frozen in 2-methylbutan at −40° C, and stored in liquid nitrogen. Frozen tissue pieces were embedded in Jung Tissue Freezing Medium (Leica Microsystems, Nussloch, Germany). Tissue cryosections (10 µm) were mounted on SuperFrostPlus slides (Thermo Scientific). Prior to staining, sections were warmed to room temperature, incubated in Dulbecoos's phosphate-buffered saline and dried. **[^{64}Cu]Cu-N$_2$py$_4$-MAB193** at a fixed concentration (10 pM) was preincubated with the competitor anti-BCMA sdAb **269A37948** at increasing concentrations (0.1−1 nM, corresponding to a 10−100-fold molar excess) in binding buffer (Dulbecco's phosphate-buffered saline containing 2.5% (w/v) bovine serum albumin) for 20 min at room temperature. Tissue cryosections were incubated with the **[^{64}Cu]Cu-N$_2$py$_4$-MAB193**/sdAb solutions for 90 min in humidified chambers, washed 2 times for 5 min in binding buffer, dipped in distilled water, and dried. A BAS-SR phosphoimaging plate was exposed to the tissue sections for 45 min in the dark and measured using an Amersham Typhoon™ FLA 9500 (Cytiva). Images were analyzed using AIDA version 5.1 (Elysia-raytest).

Flow cytometry: Fc receptor levels of myeloma and melanoma cells

General flow cytometry analysis is described above. We used the following primary antibodies: monoclonal mouse anti-human Fcγ RI/CD64 antibody, Clone #10.1, anti-human Fcγ RII/CD32, Clone 190723, anti-human CD23/Fcε RII, Clone 138628, or anti-human Fc γ RIII/CD16, Clone 245536 (all Bio-Techne). Human PBMC were used as positive control and were separated from freshly obtained heparinized human blood using LeucosepTM tubes (Greiner Bio-One, Kremsmünster, Austria) according to the manufacturer´s instructions.

II. Binding of mAbs to recombinant BCMA

Binding kinetics and binding constants: Human BCMA

SPR measurements showed that His-tagged BCMA could successfully be captured of FC(active) by the immobilized anti-His antibody (Figure S 2 A) time interval at ~0–50 s). To compensate from drift resulting from dissociation of His-tagged BCMA from the surface, a stabilization time of 420 s was included to wait for equilibration as expressed by lowered baseline drift. The drift however could not be completely avoided during the subsequent analysis as visible for the performed blank runs (Figure S 2 , time interval at ~500–3100 s), which was however regarded to be of minor and acceptable impact and in part compensated by the inclusion of blank runs to obtain double-referenced sensorgrams (Figure S 2 B).

During the single-cycle kinetic for anti-human BCMA mAbs **MAB193** and **Vicky-1** a clear and concentration dependent binding signal (Figure S2A, red and blue, respectively, time interval at ~500–2200 s) as well as a decrease of binding signal resulting from dissociation of anti-human BMCA mAb from captured human BCMA in the dissociation phase (Figure S 2 A, time interval at ~2200 – 3100 s) could be detected. In contrast, **E6D7B** showed no binding signal in this setup as visible from sensorgrams comparable to the blank runs (Figure S 2 A). Of note, mAbs **MAB193** and **Vicky-1** showed binding only to FC(active) having captured human-BCMA but not to FC(reference) where no human-BCMA was present on the surface (data not shown), which therefore could serve as suitable reference FC.

Single-cycle kinetic analysis of double-referenced sensorgrams (Figure S 2 B) showed a similar binding affinity for both **MAB193** and **Vicky-1** (K_d values 0.33 nM and 0.38 nM, respectively). The mAb-BCMA-complexes formed by **MAB193** and **Vicky-1** showed similar dissociation half-lives (42 min and 52 min, respectively). Additional binding parameters are summarized in Table S 1.

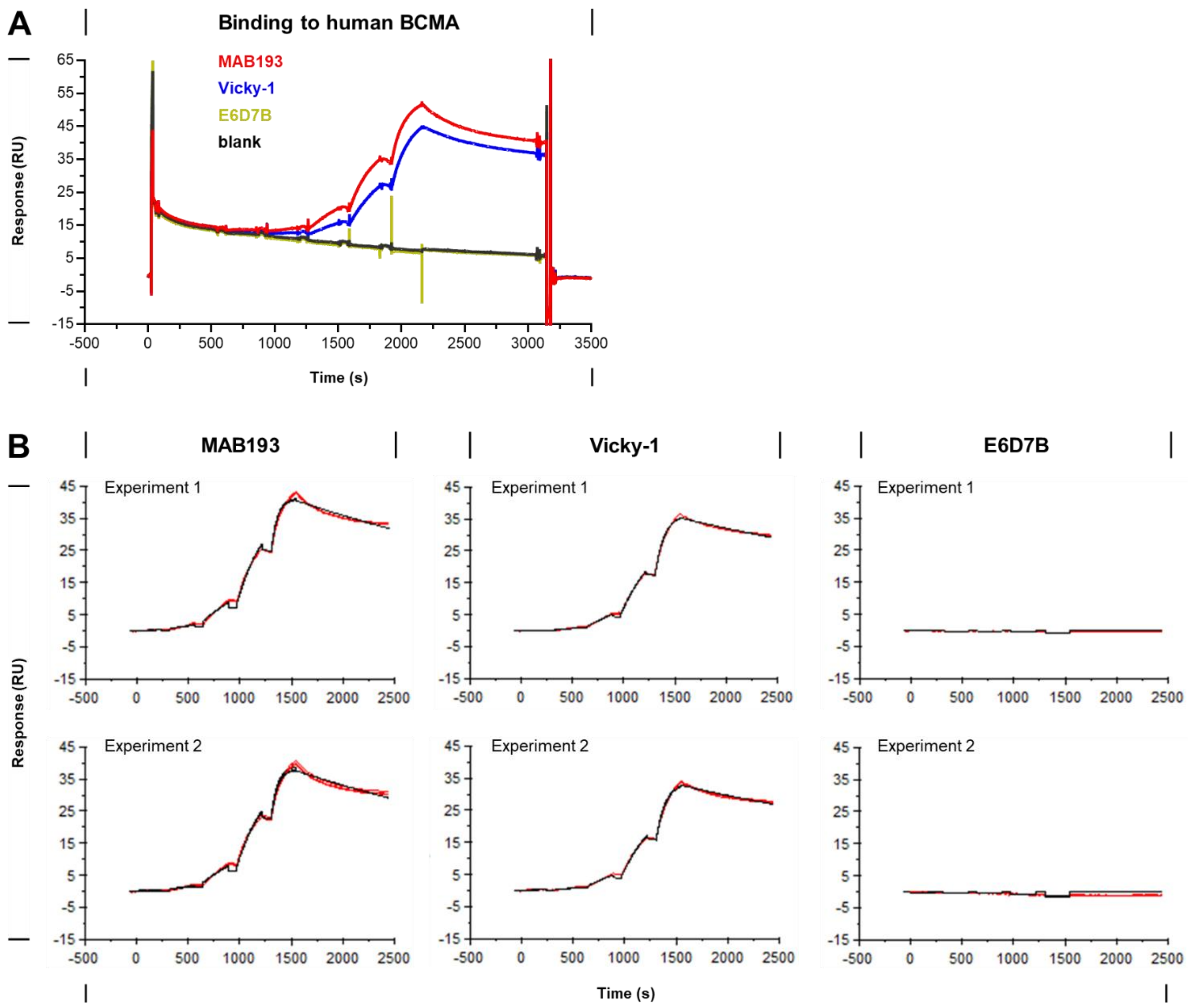

Figure S 2: (A) Sensorgrams showing the SPR responses from single-cycle-kinetic analyses of mAbs (0.032–20 nM) to captured His-tagged human BCMA (captured at 0.25–0.5 µg/mL) on the anti-His antibody-functionalized CM5 chip; **MAB193** (red), **Vicky-1** (blue), and **E6D7B** (yellow); The same sequence with buffer (HBS-P+) instead of mAbs was included as blank (black) to obtain double-referenced sensorgrams; (B) Double-referenced sensorgrams (red) analyzed by curve fitting with a 1:1 binding model (black); data obtained from two independent experiments each performed in triplicate; (RU) resonance units.

Table S 1: Rate and affinity constants for binding of mAbs to His-tagged human BCMA calculated from SPR sensorgrams; (k_a) association rate constant, (k_d) dissociation rate constant, (K_d) dissociation constant; (SE)°standard error as obtained from the instrument; (RU) resonance units; (SEM)°standard error of the mean; results from analyses of two independent experiments each performed in triplicate

Antibody		k_a (1/Ms)	*SE* (k_a)	k_d (1/s)	*SE* (k_a)	K_d (M)	SEM	RUmax	Chi² (RU²)
**MAB193**		847000	7.9E2	0.000266	5.1E-7	3.14E−10		41.3	0.661
		825000	8.3E2	0.000283	5.5E-7	3.43E−10		38.4	0.637
	**mean**	**836000**		**0.000275**		**3.29E−10**	1.45E-11		
**Vicky-1**		567000	3.5E2	0.000213	2.8E-7	3.76E−10		37.2	0.159
		563000	4.1E2	0.000223	3.3E-7	3.97E−10		34.5	0.185
	**mean**	**565000**		**0.000218**		**3.87E−10**	1.05E-11		
**E6D7B**						no binding			

Species-selectivity: Human versus murine BCMA

Species selectivity of anti-human BCMA mAbs for His-tagged human BCMA was investigated in a separate SPR experiment in direct comparison with His-tagged murine BCMA. The responses demonstrate the selectivity of **MAB193** and **Vicky-1** for binding to human BCMA over murine BCMA (Figure S 3). Although **MAB193** showed some residual binding to murine BCMA, the response at the highest antibody concentration was still very low (2.3 RUmax) compared to the response from capturing human BCMA (152 RUmax). Of note, similar levels of both murine BCMA and human BCMA were present on FC(active) at this time point (blanks: ~23 RU and ~16 RU, respectively). Since the responses indicate reactivity of **MAB193** with only a minor fraction of murine BCMA (less than 5%), yet this was interpreted as 'non-cross-reactive'. **Vicky-1** showed no binding to murine BCMA but potent binding to human BCMA (118 RUmax). **E6D7B** showed no binding to murine BCMA or human BCMA. **Anti-mouse BCMA-IgG** showed potent binding to murine BCMA (100 RUmax), but not to human BCMA, confirming the validity of the experimental setup.

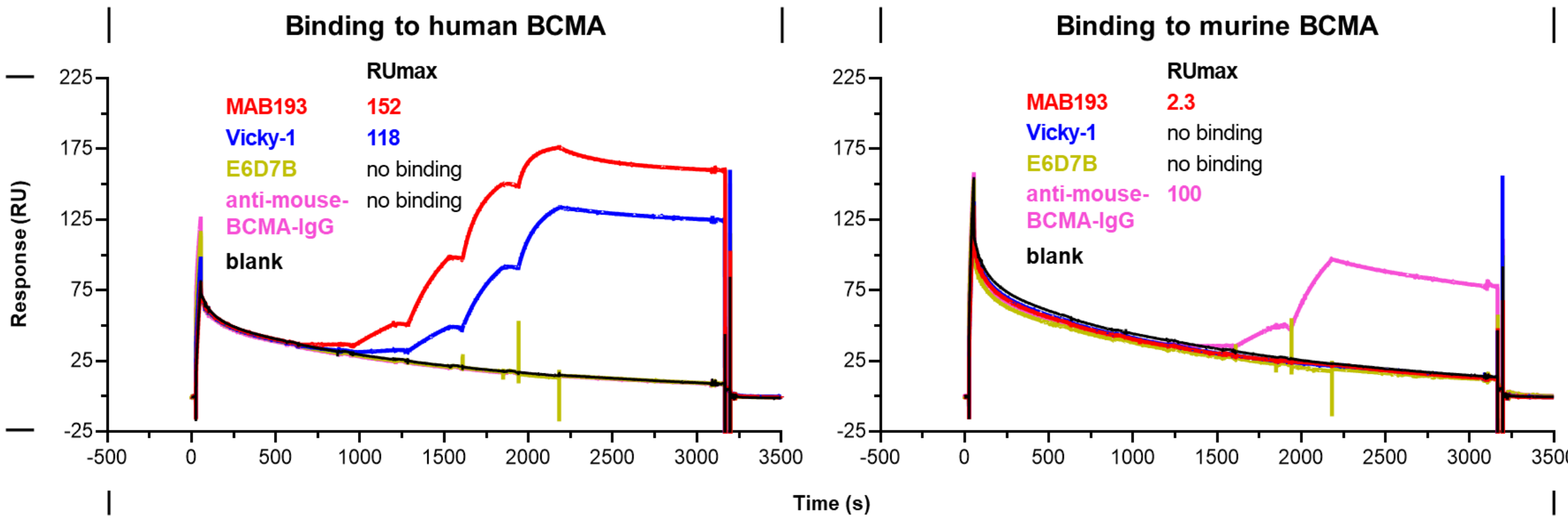

Figure S 3: Sensorgrams showing SPR responses from single-cycle kinetic analysis of mAbs (0.08–50 nM) to captured His-tagged human BCMA (captured at 0.5 µg/mL) or murine BCMA (captured at 1 µg/mL) on an anti-His antibody-functionalized CM5 chip; **MAB193** (green), **Vicky-1** (blue), and **E6D7B** (red), and **anti-mouse BCMA-IgG** (magenta); The same sequence measured with buffer instead of mAbs only was included as blank (black); Maximum binding responses (RUmax) from curve fitting of double-referenced sensorgrams with a 1:1 binding model; Responses < 1 RU at the highest antibody concentration are reported as 'no binding'

III. Bioconjugation of mAbs with a bispidine chelator

Chemical properties of bispidine-mAb conjugates

The functionalized hexadentate bispidine chelator **N_2py_4-Bn-NCS** was synthesized according to the literature [35, 37, 41]. For functionalization of the anti-human BCMA mAbs **MAB193** and **Vicky-1** as well as the mAb isotype control **IgG_{2A}-ITC** with N_2py_4, an aromatic isothiocyanate group was introduced at the C9-position of the N_2py_4 ligand forming a thiourea bond [37, 89]. The degree of conjugation after this isothiocyanate-mediated coupling using a 10-fold molar excess of **N_2py_4-Bn-NCS** was determined by MALDI-TOF MS analysis (Figure S 4 A–C) resulting in conjugate-specific values for **N_2py_4-MAB193** (1.0), **N_2py_4-Vicky-1** (0.5), and **N_2py_4-IgG_{2A}-ITC** (0.7).

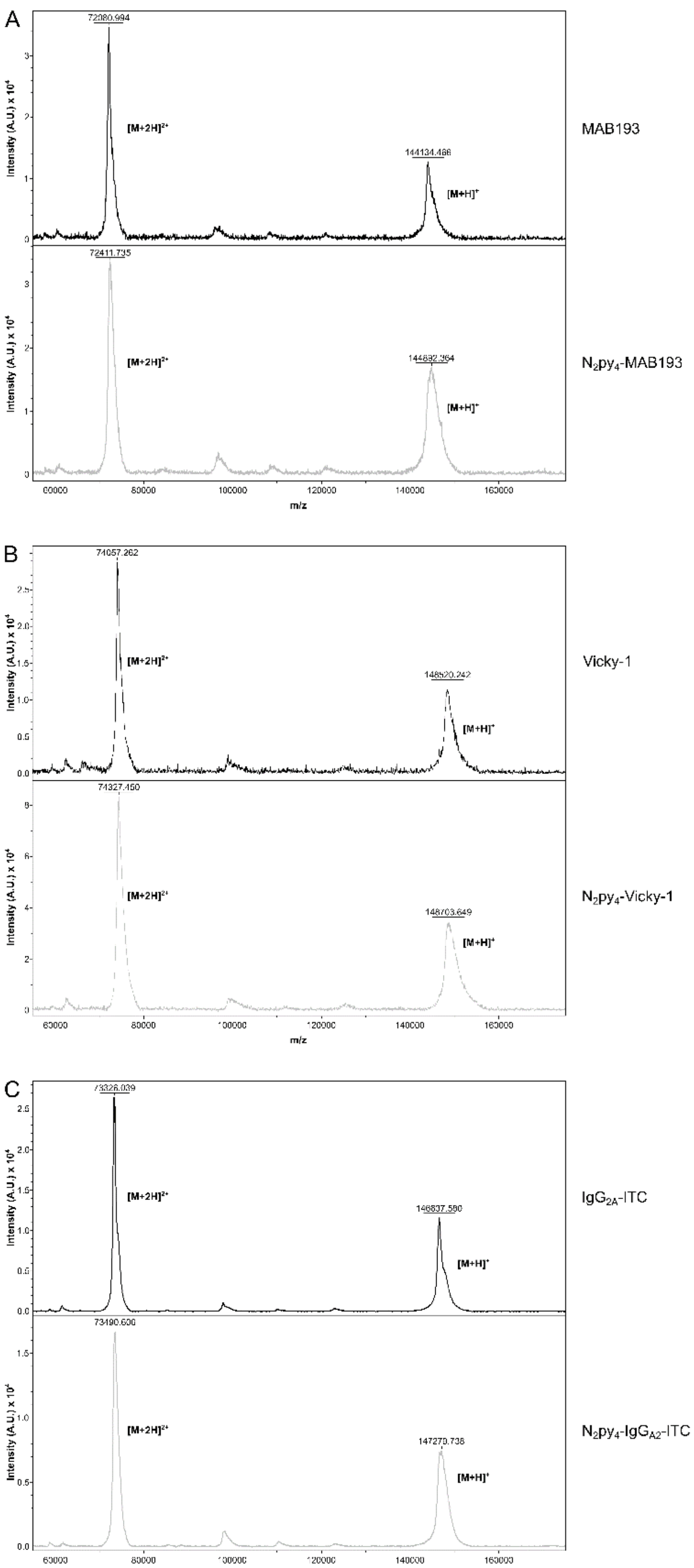

Figure S 4: MALDI-TOF mass spectra of mAbs **MAB193** (A), **Vicky-1** (B) and **IgG$_{2A}$-ITC** (C) before and after modification with a 10-fold molar excess of **N$_2$py$_4$-Bn-NCS** showing the singly $[M+H]^+$ and doubly $[M+2H]^{2+}$ charged ions

IV. Cellular binding of mAbs and bispidine-mAb conjugates

Binding constants for mAbs and bispidine-mAb conjugates towards BCMA-positive myeloma cells

Using flow cytometry analyses, it was possible to compare the binding of three mAbs to BCMA on human myeloma cell lines. The fluorescence intensities of both U266 and L363 cells (NC, negative controls incubated with secondary antibody only) increased markedly stronger after incubation with **MAB193,** and **Vicky-1** compared to **E6D7B** (Figure S 5 A–B). This indicates that **MAB193** and **Vicky-1** specifically recognize BCMA on human myeloma cells, also confirming the trends from *in vitro* binding to recombinant human BCMA (see also *Supporting information*, section I). However, **E6D7B** was excluded from further investigations due to insufficient target binding.

As exemplified by **MAB193**, the mAb selectively recognized the BCMA-positive myeloma cell lines U266 and L363, but not the BCMA-negative melanoma cell line A375 (Figure S 5 C). Furthermore, the fluorescence intensities of both U266 and L363 cells increased markedly stronger after incubation with **MAB193** compared to its corresponding isotype control **IgG_{2A}-ITC** (Figure S 5 D). This further supports that the recognition of myeloma cells by **MAB193** is BCMA-specific. After covalent modification with the bispidine chelator, the capacity of **N_2py_4-MAB193** and **N_2py_4-Vicky-1** for recognizing BCMA on U266 cells remained unchanged (Figure S 5 E–F), providing the rationale to further investigate the radiocopper-labeled bispidine-mAb conjugates.

Flow cytometry analysis of myeloma cells exposed to increasing concentrations of anti-human BCMA mAbs allowed the calculation and comparison of their binding constants (Figure S 5 G–H). The fluorescence intensities from binding of **MAB193** and **Vicky-1** showed that the BCMA concentration was 6.8–8.3-fold higher in U266 cells compared to L363 cells. Both mAbs recognized BCMA on myeloma cells with binding affinities in the low nanomolar range, specifically 10.4–23.3 nM for **MAB193** and 4.01–6.48 nM for **Vicky-1**. A375 melanoma cells showed no BCMA signal and were therefore used as negative controls.

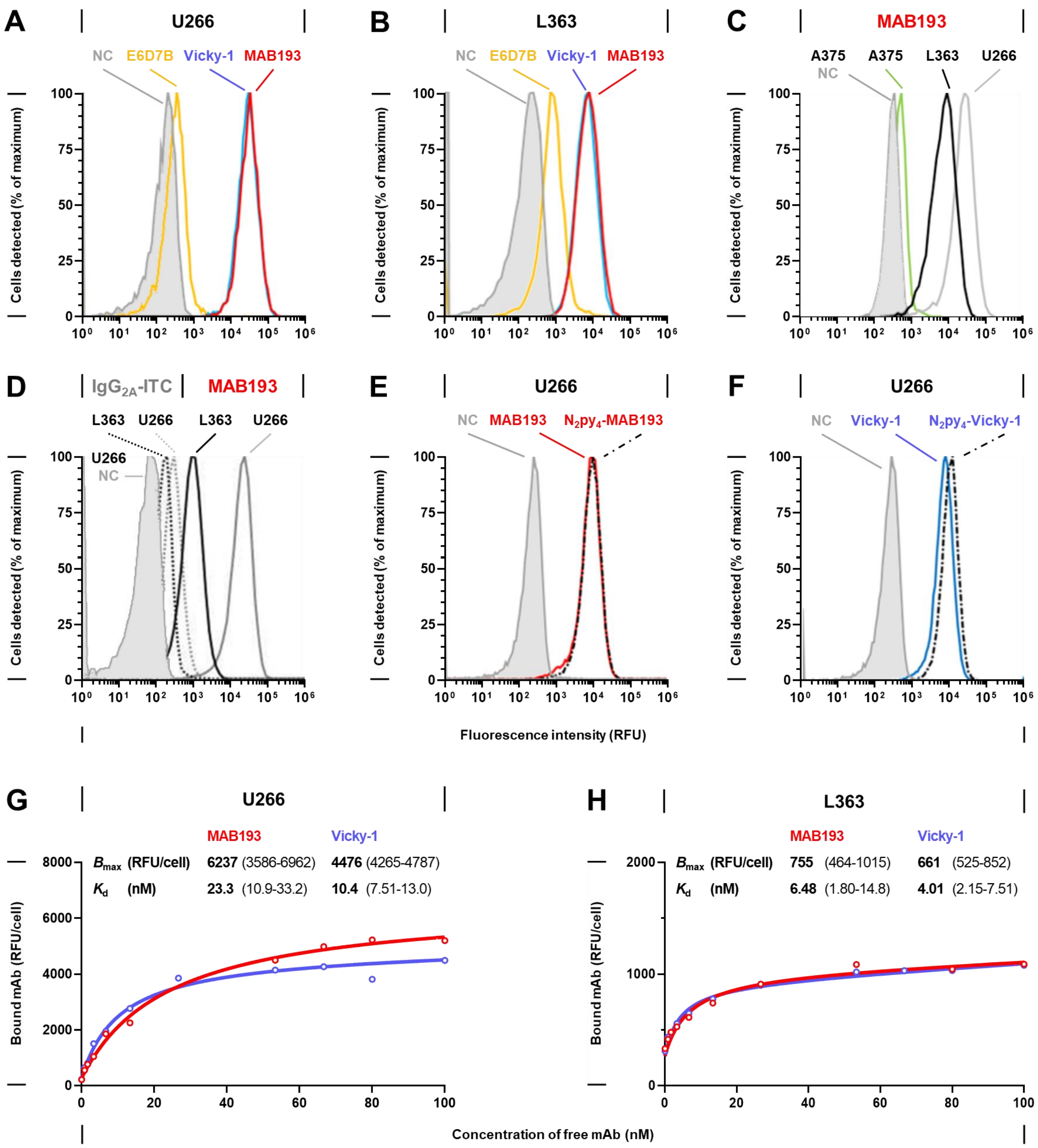

Figure S 5: Examples of flow cytometry analyses showing selective binding of **MAB193** and **Vicky-1** to myeloma cells depending on their BCMA levels; (A, B) Higher capacity for binding of **MAB193** (red) and **Vicky-1** (blue) compared to **E6D7B** (yellow) towards BCMA-positive U266 and L363 myeloma cells; (C, D) Higher capacity for binding of **MAB193** towards U266 cells (gray) and L363 cells (back) cells compared to BCMA-negative A375 melanoma cells (green) or the corresponding IgG_{2A} isotype control (ITC, dotted lines); (E, F) Capacities of **MAB193** (red) and **Vicky-1** (blue) for binding to U266 cells remained unchanged after covalent modification with the bispidine chelator N_2py_4 (dot-dash lines); (G, H) Higher binding capacity (B_{max}) of U266 compared to L363 cells for both **MAB193** (red) and **Vicky-1** (blue); binding affinities (K_d) in the low nanomolar range; (NC) negative control incubated with secondary antibody only, (RFU) relative fluorescence units

V. Radiolabeling of bispidine-mAb conjugates

Radiochemical properties of copper-64 and copper-67-labeled N_2py_4-mAb conjugates

Radiolabeling of the bispidine-mAb conjugates with copper-64 at molar activities between 50 and 80 MBq/nmol resulted in preparations of **[^{64}Cu]Cu-N_2py_4-MAB193**, **[^{64}Cu]Cu-N_2py_4-Vicky-1,** and **[^{64}Cu]Cu-N_2py_4-IgG$_{2A}$-ITC** with radiochemical purities of >99% (Figure S 6 A, B). The R_f values of TLC system i) are as follow: [^{64}Cu]$CuCl_2$, [^{64}Cu]Cu-N_2py_4-Ab = 0, [^{64}Cu]Cu-N_2py_4 ~0.6 and of TLC system ii): [^{64}Cu]Cu-N_2py_4-Ab = 0, [^{64}Cu]Cu-N_2py_4, [^{64}Cu]$CuCl_2$ ~0.9–1. Radioluminographic imaging of gel electrophoresis of the bispidine-mAb conjugates showed predominant radiolabeling of the heavy chain, indicating a preceding preferential modification of this particular structural element with the chelator (Figure S 6 C). Furthermore, most copper-64-labeled bispidine-mAb conjugates contained a single chelator unit linked to the heavy chain, whereas the light chain remained largely unmodified.

The bispidine-mAb conjugates were radiolabeled with copper-67 in a similar manner, resulting at molar activities of 30–50 MBq/nmol with radiochemical purities of >99%.

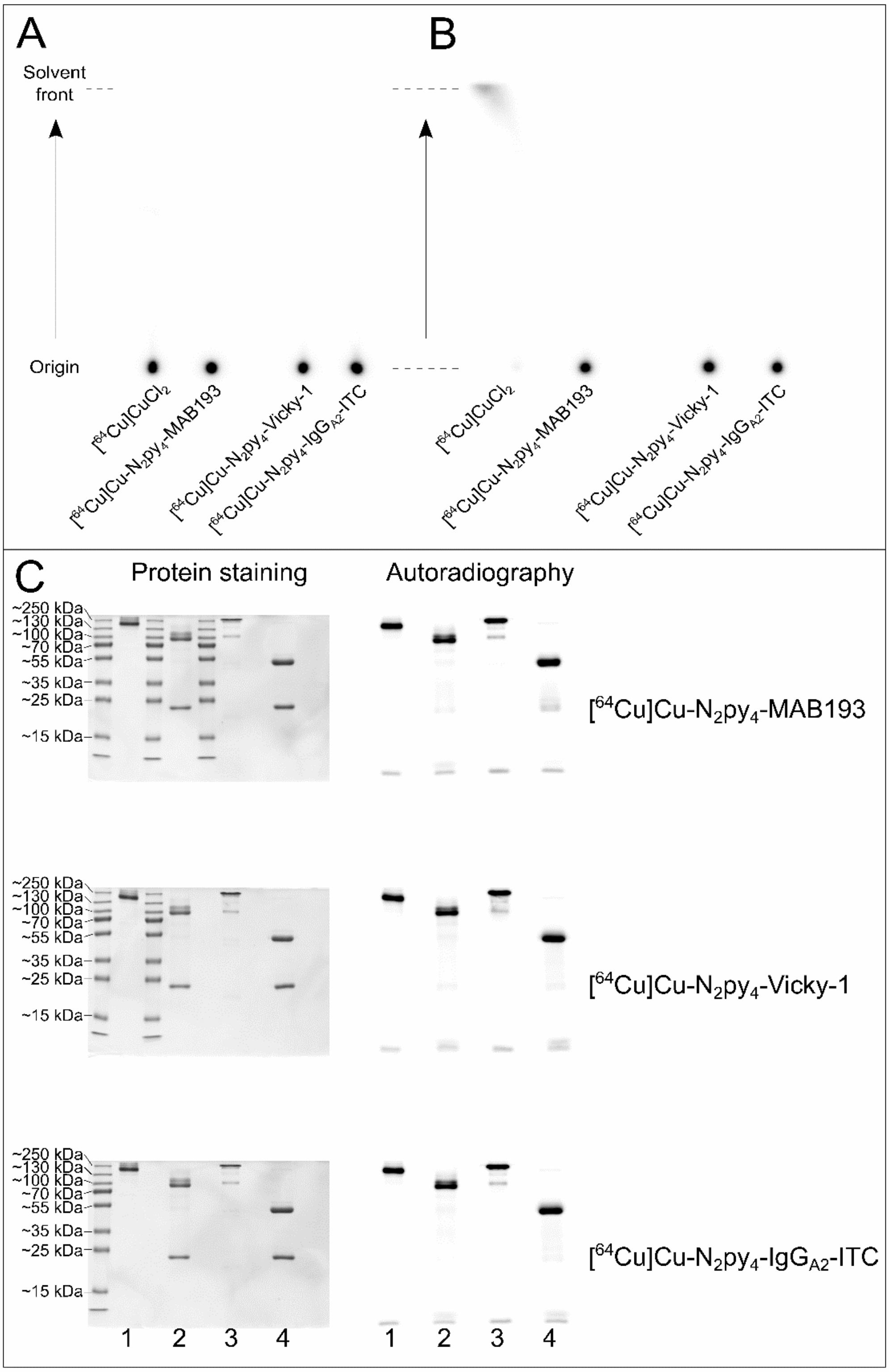

Figure S 6: Radiolabeling of bispidine-mAb conjugates. **(A, B)** Radio-TLC analysis of the bispidine-mAb conjugates labeled with copper-64 and analyzed by radio-TLC using either **(A)** neutral alumina plates coated with silica gel and acetonitrile/H_2O (70:30, v/v) or **(B)** glass microfiber chromatography paper impregnated with silica gel (iTLC-SG) and 50 mM EDTA (aq.; pH 5.5); **(C)** SDS-PAGE analysis of ^{64}Cu-labeled bispidine-mAb conjugates; Coomassie stain and radioluminographic image of a 12% SDS-polyacrylamide gel showing protein bands corresponding to the molecular weights of the intact IgG (~150 kDa), the monomeric light chain (~24 kDa) and heavy chain (~50 kDa) chain as well as to the dimeric heavy chain (~100 kDa); lane 1: samples not reduced or denatured before electrophoresis, lane 2: samples reduced before gel loading, lane 3: samples heat-denatured prior to electrophoretic separation, lane 4: samples reduced as well as heat-denatured before electrophoresis.

VI. Distribution of copper-64-labeled anti-BCMA mAbs *in vivo*

[^{64}Cu]Cu-N$_2$py$_4$-Vicky-1: PET images and image-derived uptake values in U266 xenograft mice

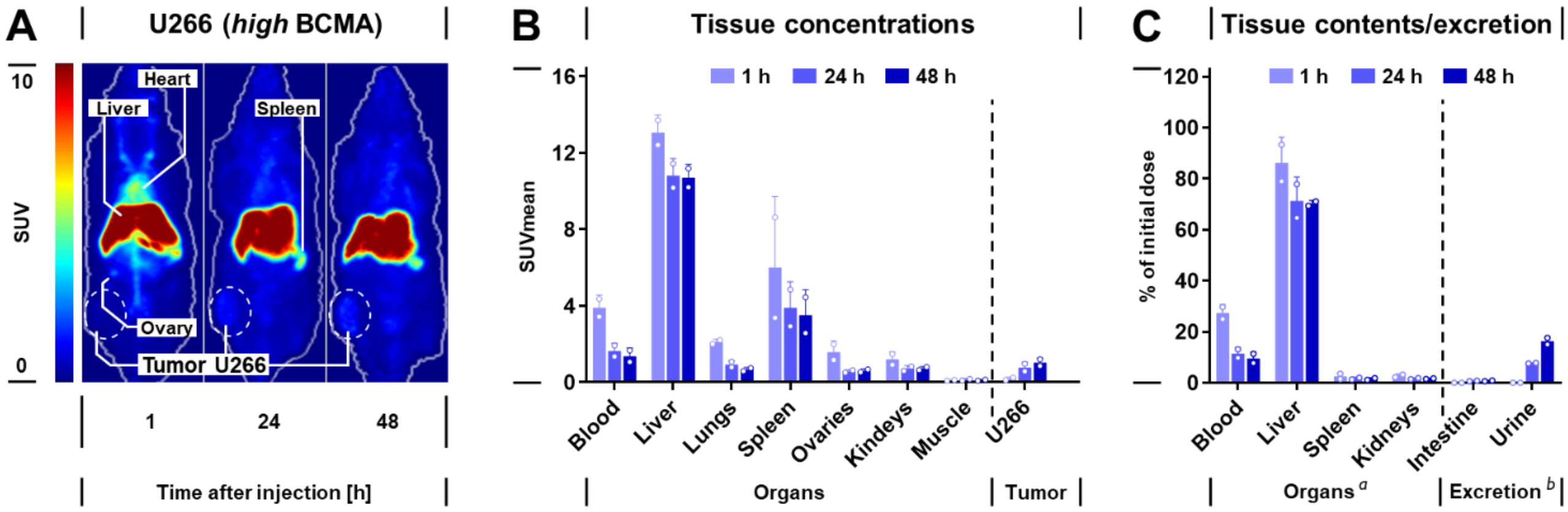

Figure S 7: PET imaging and image-derived uptake values showing the tissue-specific distribution of **[^{64}Cu]Cu-N$_2$py$_4$-Vicky-1** in mice bearing subcutaneous U266 myeloma xenografts; (A) PET images at indicated time points; maximum intensity projections; (B) Biological concentrations in organs and tumors extracted from quantitative analysis of PET images; (C) Tissue-specific contents: [a] extrapolated from biological concentrations using a realistic dose model for 30 g-mice, [b] calculated through balancing contents measured in intestine, urinary bladder, and total body; see materials and methods for details; (SUVmean) region-averaged standardized uptake value; means ± standard deviation

[^{64}Cu]Cu-N$_2$py$_4$-MAB193 versus [^{64}Cu]Cu-N$_2$py$_4$-Vicky-1: Ex-vivo uptake values in tissue explants from U266 xenograft mice

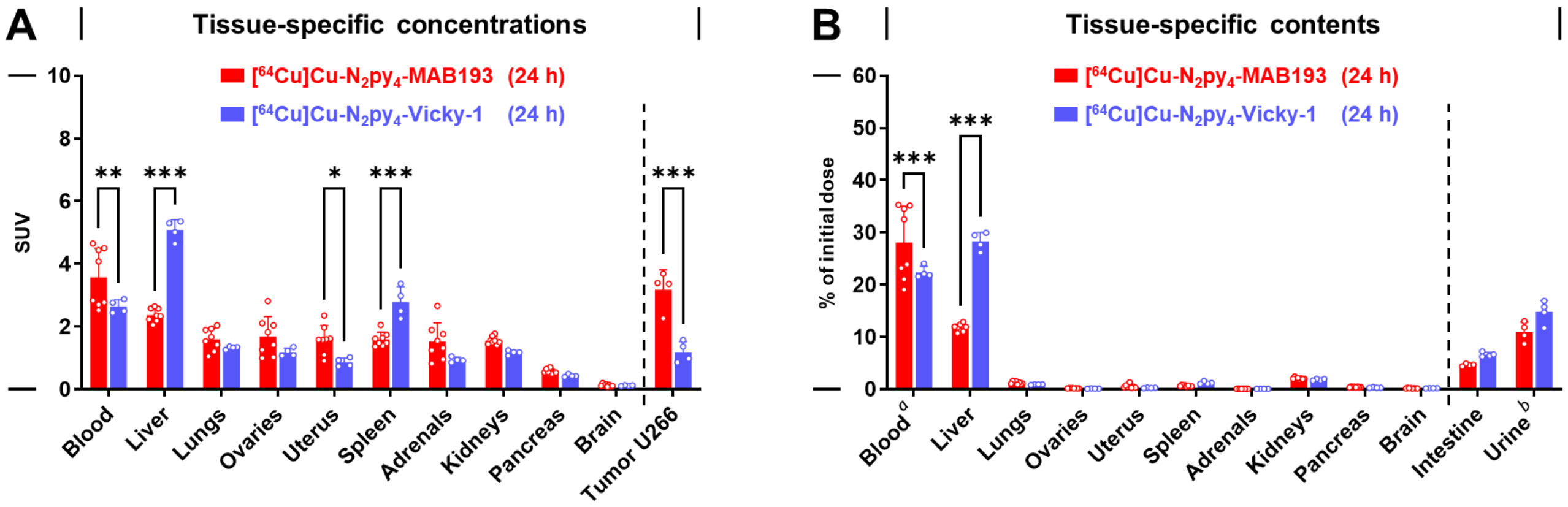

Figure S 8: Distribution of **[^{64}Cu]Cu-N$_2$py$_4$-MAB193** and **[^{64}Cu]Cu-N$_2$py$_4$-Vicky-1** measured *ex vivo* in tissue explants prepared from nude mice bearing subcutaneous U266 myeloma xenografts; tissues were excised 24 h after injection of the radiolabeled mAbs; (A) Mass-averaged uptake values in organs and tumors calculated from decay-corrected activity in tissue samples; (B) Total tissue-specific contents: [a] extrapolated from mass-averaged uptake values using a realistic dose model for 30 g-mice, [b] calculated through balancing contents measured in intestine, urinary bladder, and total body; (SUV) standardized uptake value; data presented as means ± standard deviation; significance of differences: * $p < 0.05$, ** $p < 0.01$; *** $p < 0.001$

[^{64}Cu]Cu-N$_2$py$_4$-MAB193 *versus* ***[^{64}Cu]Cu-N$_2$py$_4$-Vicky-1****: Radioluminographic analysis of uptake in U266 tumors*

Uptake of copper-64-labeled bispidine-mAb conjugates was examined *ex vivo* in tissue explants of subcutaneous U266 xenograft mouse models. Radioluminograms of tissue cryosections showed specific accumulation of the bispidine-mAb conjugates in tumors, but not in muscle. **[^{64}Cu]Cu-N$_2$py$_4$-MAB193** showed a 2-fold higher tumor-to-muscle ratio compared to **[^{64}Cu]Cu-N$_2$py$_4$-Vicky-1** (Figure S 9).

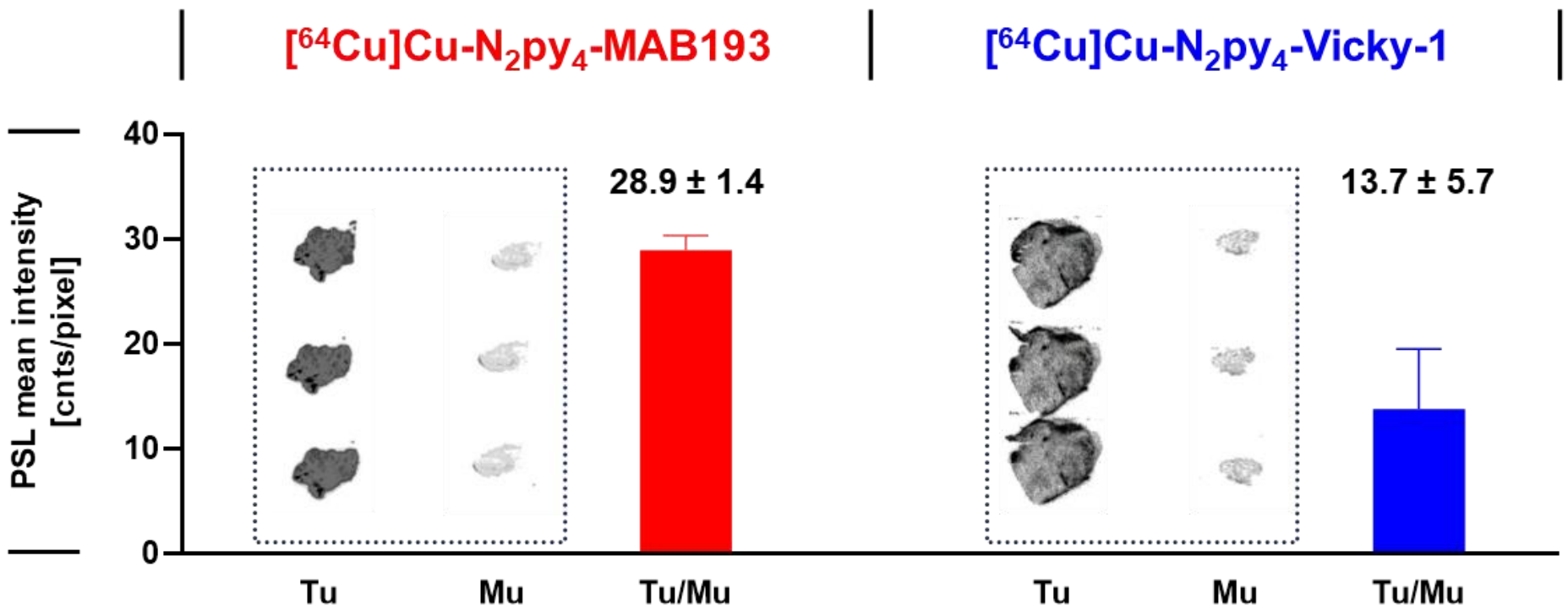

Figure S 9: Tumor-specific uptake of **[^{64}Cu]Cu-N$_2$py$_4$-MAB193** and **[^{64}Cu]Cu-N$_2$py$_4$-Vicky-1** in subcutaneous U266 myeloma xenografts measured *ex vivo* in tissue explants 24 h after injection of the radiolabeled mAbs; (PSL intensity) photostimulated luminescence intensity; dotted regions show tissue-specific radioluminograms (with common PSL intensity scale) included in quantitative image analysis; (cnts) counts; (Mu) muscle, (Tu) tumor, (Tu/Mu) tumor-to-muscle ratio of PSL intensities; means ± standard deviation (n = 3)

[^{64}Cu]Cu-N$_2$py$_4$-MAB193: *PET image-derived uptake values in tumor-bearing mice*

Table S 2: PET image-derived standardized uptake values and ratios of **[^{64}Cu]Cu-N$_2$py$_4$-MAB193** in nude mice bearing subcutaneous myeloma (U266, L363), or melanoma (A375) xenografts; means ± standard deviation; organs (n = 15); U266 tumors (n = 10); L363 tumors (n = 3); A375 tumors (n = 2); significance of differences compared to organs: [#] $p < 0.05$, [##] $p < 0.01$, [###] $p < 0.001$ and compared to L363 tumors: [&] $p < 0.05$, [&&] $p < 0.01$, [&&&] $p < 0.001$

**[^{64}Cu]Cu-N$_2$py$_4$-MAB193**	**ROI name**	**Uptake**			**Fold-change**
		**1 h**	**24 h**	**48 h**	**1–48 h**
SUVmean	Blood (Heart)	9.81 ± 0.68	3.92 ± 0.61	3.35 ± 0.67	0.34 ± 0.07
	Liver	4.29 ± 0.47	2.85 ± 0.35	2.72 ± 0.38	0.63 ± 0.09
	Lungs	3.71 ± 0.45	2.02 ± 0.19	1.71 ± 0.15	0.46 ± 0.04
	Spleen	3.08 ± 0.61	1.65 ± 0.45	1.54 ± 0.42	0.50 ± 0.14
	Ovaries	3.99 ± 2.12	1.66 ± 0.43	1.54 ± 0.44	0.39 ± 0.11
	Kidneys	2.76 ± 0.31	1.44 ± 0.09	1.21 ± 0.14	0.44 ± 0.05
	Muscle	0.24 ± 0.04	0.29 ± 0.03	0.27 ± 0.04	1.12 ± 0.19
	**Tumor U266**	**0.53 ± 0.18**[###]	**2.74 ± 0.69**[&&]	**4.01 ± 0.87**[###,&&&]	**7.64 ± 1.65**[###]
	**Tumor L363**	**0.32 ± 0.18**[##]	**1.71 ± 0.17**	**2.59 ± 0.76**	**8.00 ± 2.35**[###]
	**Tumor A375**	**0.54 ± 0.15**[##]	**1.96 ± 0.06**	**2.56 ± 0.41**	**4.79 ± 0.77**[###,&]
SUVmean Tissue-to-Blood ratio	Liver	0.43 ± 0.04	0.74 ± 0.08	0.83 ± 0.12	1.93 ± 0.27
	Lungs	0.39 ± 0.04	0.53 ± 0.06	0.53 ± 0.08	1.38 ± 0.20
	Spleen	0.32 ± 0.05	0.43 ± 0.10	0.48 ± 0.11	1.51 ± 0.34
	Ovaries	0.41 ± 0.19	0.43 ± 0.11	0.47 ± 0.12	1.15 ± 0.31
	Kidneys	0.42 ± 0.05	0.71 ± 0.11	0.80 ± 0.18	1.90 ± 0.42
	Muscle	0.02 ± 0.01	0.08 ± 0.01	0.08 ± 0.01	3.44 ± 0.59
	**Tumor U266**	**0.05 ± 0.02**	**0.75 ± 0.20**[&]	**1.28 ± 0.30**[&&&]	**23.5 ± 5.50**[###]
	**Tumor L363**	**0.03 ± 0.02**	**0.37 ± 0.06**	**0.68 ± 0.25**	**22.8 ± 8.38**[###]
	**Tumor A375**	**0.06 ± 0.01**	**0.56 ± 0.02**	**1.11 ± 0.16**[&]	**18.5 ± 2.59**[###]
SUVmean Tissue-to-Muscle ratio	Liver	18.4 ± 3.69	9.88 ± 1.71	10.3 ± 2.07	0.56 ± 0.11
	Lungs	16.5 ± 3.84	7.04 ± 1.16	6.50 ± 0.96	0.39 ± 0.06
	Spleen	13.5 ± 2.63	5.73 ± 1.46	5.89 ± 1.42	0.44 ± 0.11
	Ovaries	16.9 ± 8.35	5.58 ± 1.16	5.66 ± 1.38	0.34 ± 0.08
	Kidneys	18.0 ± 4.41	9.49 ± 2.32	9.89 ± 2.73	0.55 ± 0.15
	**Tumor U266**	**2.22 ± 0.80**[###]	**9.26 ± 2.71**	**14.9 ± 3.61**[###,&&]	**6.71 ± 1.63**[###]
	**Tumor L363**	**1.41 ± 0.82**[###]	**6.29 ± 1.15**	**9.53 ± 0.61**	**6.77 ± 0.44**[##]
	**Tumor A375**	**1.71 ± 0.04**[###]	**6.26 ± 1.28**	**8.45 ± 1.55**	**4.94 ± 0.91**

[^{64}Cu]Cu-N$_2$py$_4$-IgG$_{2A}$-ITC *(MAB193 isotype control): PET image-derived uptake values in tumor-bearing mice*

Table S 3: PET image-derived standardized uptake values and ratios of the mAb isotype control **[^{64}Cu]Cu-N$_2$py$_4$-IgG$_{2A}$-ITC** in mice bearing myeloma (U266, L363), or melanoma (A375) xenografts; means ± standard deviation; organs (n = 13); U266 tumors (n = 8); L363 tumors (n = 2); A375 tumors (n = 3); significance of differences compared to **[^{64}Cu]Cu-N$_2$py$_4$-MAB193** (ref. Table S 2): § $p < 0.05$, §§ $p < 0.01$, §§§ $p < 0.001$

**[^{64}Cu]Cu-N$_2$py$_4$-IgG$_{2A}$-ITC**	**ROI name**	**Uptake**			**Fold-change**
		**1 h**	**24 h**	**48 h**	**1–48 h**
SUVmean	Blood (Heart)	8.33 ± 0.48§§§	2.84 ± 0.20§§§	2.34 ± 0.17§§§	0.28 ± 0.02
	Liver	6.46 ± 0.53§§§	4.83 ± 0.48§§	5.01 ± 0.29§§§	0.78 ± 0.04
	Lungs	3.93 ± 0.37	1.70 ± 0.17	1.46 ± 0.19	0.37 ± 0.05
	Spleen	3.08 ± 0.54	1.73 ± 0.25	1.72 ± 0.27	0.56 ± 0.09
	Ovaries	2.80 ± 0.62§§	1.31 ± 0.27	1.20 ± 0.29	0.43 ± 0.10
	Kidneys	2.43 ± 0.26	1.15 ± 0.11	1.05 ± 0.09	0.43 ± 0.04
	Muscle	0.20 ± 0.03	0.29 ± 0.02	0.28 ± 0.02	1.39 ± 0.10
	**Tumor U266**	**0.40 ± 0.11**	**0.92 ± 0.23§§§**	**1.05 ± 0.22§§§**	**2.62 ± 0.54§§§**
	**Tumor L363**	**0.28 ± 0.08**	**0.76 ± 0.18**	**0.91 ± 0.28§§**	**3.29 ± 1.00**
	**Tumor A375**	**0.46 ± 0.14**	**1.94 ± 0.06**	**2.16 ± 0.06**	**4.69 ± 0.13**
SUVmean Tissue-to-Blood ratio	Liver	0.78 ± 0.05§§§	1.71 ± 0.24§§§	2.14 ± 0.16§§§	2.77 ± 0.20
	Lungs	0.47 ± 0.04	0.6 ± 0.06	0.62 ± 0.05	1.31 ± 0.10
	Spleen	0.37 ± 0.06	0.61 ± 0.07§	0.73 ± 0.13§§	1.98 ± 0.34
	Ovaries	0.34 ± 0.08	0.47 ± 0.12	0.51 ± 0.12	1.51 ± 0.35
	Kidneys	0.72 ± 0.19§§§	1.58 ± 0.53§§§	1.95 ± 0.62§§§	2.72 ± 0.86
	Muscle	0.02 ± 0.01	0.10 ± 0.01	0.12 ± 0.01	5.22 ± 0.62
	**Tumor U266**	**0.05 ± 0.01**	**0.33 ± 0.09§§§**	**0.45 ± 0.11§§§**	**9.93 ± 2.40§§§**
	**Tumor L363**	**0.04 ± 0.01**	**0.27 ± 0.08**	**0.40 ± 0.13**	**11.4 ± 3.64**
	**Tumor A375**	**0.08 ± 0.02**	**0.86 ± 0.05**	**1.13 ± 0.11**	**13.5 ± 1.36**
SUVmean Tissue-to-Muscle ratio	Liver	33.3 ± 5.81§§§	16.7 ± 2.31§§§	18.3 ± 1.70§§§	0.55 ± 0.05
	Lungs	20.2 ± 3.70§	5.82 ± 0.44	5.31 ± 0.70	0.26 ± 0.03
	Spleen	15.8 ± 3.81	5.97 ± 0.81	6.21 ± 0.80	0.39 ± 0.05
	Ovaries	14.5 ± 4.23	4.52 ± 0.98	4.39 ± 1.22	0.30 ± 0.08
	Kidneys	30.4 ± 9.09§§§	15.5 ± 5.13§§§	16.8 ± 5.46§§§	0.55 ± 0.18
	**Tumor U266**	**1.95 ± 0.58**	**3.16 ± 0.74§§§**	**3.82 ± 0.90§§§**	**1.95 ± 0.46§§§**
	**Tumor L363**	**1.51 ± 0.23**	**2.62 ± 0.52**	**3.33 ± 0.76**	**2.21 ± 0.51**
	**Tumor A375**	**2.81 ± 0.3**	**7.92 ± 1.12**	**8.61 ± 1.16**	**3.06 ± 0.41**

$[^{64}Cu]Cu$-N_2py_4-MAB193: *Predicted human absorbed and effective doses*

Table S 4: Human absorbed and effective doses predicted for PET imaging using **$[^{64}Cu]Cu$-N_2py_4-MAB193**; calculated from projected human time-activity courses extrapolated from quantitative PET imaging of female nude mice (*n* = 13) injected with 10–15 MBq of **$[^{64}Cu]Cu$-N_2py_4-MAB193**; number of nuclear conversions input: heart contents, kidneys, liver, spleen, ovaries, urinary bladder contents, remainder (excluding tumors); means ± standard deviation

Target organ	Women (60 kg)	Men (73 kg)
**Absorbed doses [µGy/MBq]**		
Adrenals	41.6 ± 2.40	34.0 ± 2.00
Brain	26.7 ± 0.50	21.3 ± 0.45
Breasts	27.5 ± 0.75	–
Esophagus	35.6 ± 1.75	29.5 ± 1.40
Eyes	26.7 ± 0.50	21.4 ± 0.45
Gallbladder wall	36.7 ± 1.55	36.2 ± 2.25
Left colon	35.3 ± 1.25	27.0 ± 0.85
Small intestine	31.5 ± 0.85	26.8 ± 0.80
Stomach wall	34.4 ± 1.25	29.7 ± 1.30
Right colon	33.3 ± 0.95	28.1 ± 1.05
Rectum	31.4 ± 0.85	25.2 ± 0.70
Heart wall	138 ± 16.0	120 ± 13.0
Kidneys	70.3 ± 7.45	60.4 ± 6.35
Liver	114 ± 14.0	99.3 ± 12.5
Lungs	33.6 ± 1.30	27.2 ± 1.15
Ovaries	72.5 ± 30.2	–
Pancreas	37.9 ± 1.65	30.6 ± 1.30
Prostate	–	25.1 ± 0.75
Salivary glands	27.8 ± 0.55	23.2 ± 0.55
Red marrow	24.9 ± 0.75	20.2 ± 0.70
Osteogenic cells	30.1 ± 0.60	29.4 ± 0.65
Spleen	78.0 ± 11.1	66.2 ± 9.65
Testes	–	22.4 ± 0.50
Thymus	34.8 ± 1.45	30.2 ± 1.45
Thyroid	28.8 ± 0.70	24.2 ± 0.65
Urinary bladder wall	35.6 ± 5.60	28.3 ± 4.95
Uterus	31.6 ± 0.90	–
Total body remainder	32.9 ± 1.25	26.3 ± 1.25
**Effective dose [µSy/MBq]**	**35.3 ± 3.15**	**25.3 ± 1.65**

VII. BCMA target specificity of [^{64}Cu]Cu-N$_2$py$_4$-MAB193

MAB193 and [^{64}Cu]Cu-N$_2$py$_4$-MAB193: BCMA-specificity of binding to cells and tissue cryosections

The BCMA specificity of **MAB193** was tested through displacement by increasing molar excess of the anti-human BCMA sdAb **269A37948** used as a competitor. Exemplary flow cytometry data of individual U266 cell samples incubated with **MAB193** alone or in presence of the sdAb are shown in Figure S 10 A. Quantitative analysis showed that already a 2-fold molar excess of the sdAb resulted in 80% decrease of **MAB193** binding towards U266 cells. Further binding experiments *in vitro* using tissue explants showed a similar blockade of **[^{64}Cu]Cu-N$_2$py$_4$-MAB193** binding on tumor cryosections in presence of a 10-fold molar excess of the sdAb (Figure S 10 B). Cryosections of liver and muscle served as control for non-specific binding. These data support that binding of **MAB193** and **[^{64}Cu]Cu-N$_2$py$_4$-MAB193** to myeloma cells and xenograft tumors is BCMA-specific.

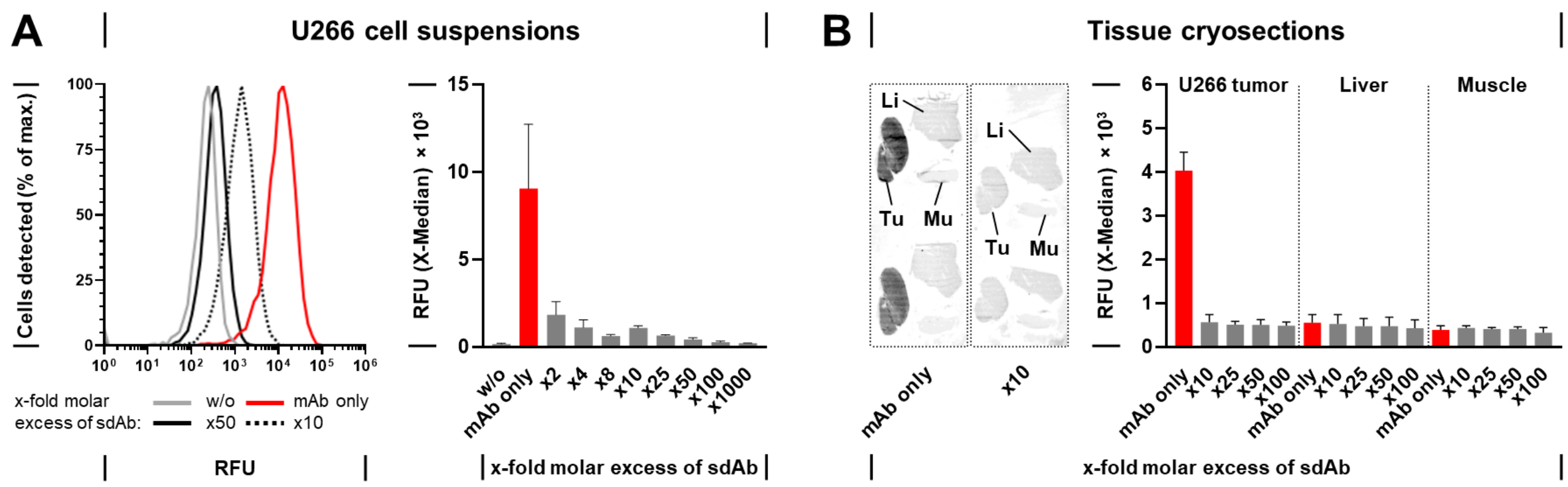

Figure S 10: BCMA-specific blockade of **MAB193** and **[^{64}Cu]Cu-N$_2$py$_4$-MAB193** binding with the anti-human BCMA sdAb **269A37948** *in vitro*; (A) Exemplary flow cytometry data and quantitative analysis of fluorescence intensities (RFU) of U266 cells incubated with **MAB193** alone or in presence of excess sdAb; two to three independent experiments; (w/o) cells incubated with secondary antibody only; (B) Exemplary radioluminograms and quantitative analysis of photostimulated luminescence (PSL) intensities of tissue sections incubated with **[^{64}Cu]Cu-N$_2$py$_4$-MAB193** alone or in presence of excess sdAb; four replicates; means ± standard deviation; (Li) liver, (Mu) muscle, (Tu) tumor

VIII. Distribution of [^{67}Cu]Cu-N_2py_4-MAB193 in vivo

[^{67}Cu]Cu-N_2py_4-MAB193: SPECT image-derived uptake values in U266 xenograft mice

Table S 5: SPECT image-derived standardized uptake values and ratios of the theranostic mAb **[^{67}Cu]Cu-N_2py_4-MAB193** in mice bearing subcutaneous U266 myeloma xenografts; means ± standard deviation; organs (*n* = 3); U266 tumors (*n* = 3); significance of differences compared to **[^{64}Cu]Cu-N_2py_4-MAB193** (ref. Table S 2): §§ $p < 0.01$

[^{67}Cu]Cu-N_2py_4-MAB193	ROI name	Uptake				Fold-change
		**1 h**	**24 h**	**48 h**	**5 d**	**1–48 h**
SUVmean	Blood (Heart)	8.51 ± 0.42	3.37 ± 0.28	2.77 ± 0.17	1.71 ± 0.37	0.20 ± 0.04
	Liver	3.72 ± 0.59	2.53 ± 0.04	2.40 ± 0.24	2.00 ± 0.27	0.54 ± 0.07
	Lungs	3.12 ± 0.34	1.70 ± 0.22	1.29 ± 0.07	0.98 ± 0.26	0.32 ± 0.08
	Spleen	3.77 ± 1.05	2.28 ± 0.29	2.34 ± 0.14	1.69 ± 0.13	0.45 ± 0.03
	Ovaries	4.71 ± 0.89	2.22 ± 0.67	1.75 ± 0.58	0.86 ± 0.54	0.18 ± 0.11
	Kidneys	2.77 ± 0.21	1.34 ± 0.15	1.09 ± 0.14	0.78 ± 0.14	0.28 ± 0.05
	Muscle	0.20 ± 0.07	0.32 ± 0.01	0.25 ± 0.03	0.16 ± 0.02	0.78 ± 0.10
	**Tumor U266**	**0.84 ± 0.24**	**3.79 ± 0.30**	**4.57 ± 0.58**	**5.48 ± 0.68**	**6.55 ± 0.81**
SUVmean Tissue-to-Blood ratio	Liver	0.38 ± 0.05	0.63 ± 0.04	0.75 ± 0.08	0.95 ± 0.23	2.49 ± 0.61
	Lungs	0.32 ± 0.02	0.42 ± 0.06	0.41 ± 0.05	0.45 ± 0.05	1.43 ± 0.14
	Spleen	0.39 ± 0.10	0.57 ± 0.1§§	0.74 ± 0.08	0.8 ± 0.15	2.05 ± 0.38
	Ovaries	0.48 ± 0.09	0.56 ± 0.19	0.54 ± 0.16	0.41 ± 0.23	0.85 ± 0.48
	Kidneys	0.37 ± 0.08	0.55 ± 0.18	0.65 ± 0.25	0.82 ± 0.43	2.25 ± 1.17
	Muscle	0.02 ± 0.01	0.08 ± 0.01	0.08 ± 0.02	0.07 ± 0.02	3.14 ± 0.65
	**Tumor U266**	**0.09 ± 0.02**	**0.94 ± 0.11**	**1.44 ± 0.27**	**2.62 ± 0.70**	**30.3 ± 8.08**
SUVmean Tissue-to-Muscle ratio	Liver	19.9 ± 5.83	7.88 ± 0.16	9.59 ± 0.61	12.8 ± 1.11	0.64 ± 0.06
	Lungs	16.7 ± 4.55	5.27 ± 0.60	5.20 ± 0.46	6.18 ± 0.88	0.37 ± 0.05
	Spleen	20.7 ± 9.60§§	7.12 ± 1.12	9.39 ± 0.65	10.8 ± 0.69	0.52 ± 0.03
	Ovaries	24.6 ± 4.15§§	6.89 ± 2.03	7.00 ± 2.06	5.32 ± 2.78	0.22 ± 0.11
	Kidneys	18.9 ± 6.29	6.82 ± 1.99	8.08 ± 2.15	10.7 ± 4.36	0.57 ± 0.23
	**Tumor U266**	**4.68 ± 2.31**	**11.8 ± 1.23**	**18.5 ± 3.94**	**35.6 ± 8.38**	**7.62 ± 1.79**

[^{67}Cu]Cu-N$_2$py$_4$-MAB193: *Predicted human equivalent and effective doses*

Table S 6: Human absorbed and effective doses predicted for treatment with **[^{67}Cu]Cu-N$_2$py$_4$-MAB193**; calculated from projected human time-activity courses (A) extrapolated from quantitative SPECT imaging of female nude mice (*n* = 3) treated with 25–80 MBq of **[^{67}Cu]Cu-N$_2$py$_4$-MAB193**; (B) extrapolated from quantitative PET imaging of female nude mice (*n* = 13) injected with 10–15 MBq of **[^{64}Cu]Cu-N$_2$py$_4$-MAB193;** number of nuclear conversions input: heart contents, kidneys, liver, spleen, ovaries, urinary bladder contents, remainder (excluding tumors); means ± standard deviation

Target organ	Women (60 kg)		Men (73 kg)	
**Absorbed doses [μGy/MBq]**	A	B	A	B
Adrenals	171 ± 2.08	165 ± 8.00	139 ± 2.52	137 ± 6.50
Brain	129 ± 2.65	125 ± 3.00	104 ± 2.08	101 ± 2.60
Breasts	128 ± 2.65	124 ± 4.00	–	–
Esophagus	151 ± 2.31	145 ± 6.00	127 ± 2.08	123 ± 5.00
Eyes	129 ± 2.65	125 ± 3.00	104 ± 2.08	101 ± 2.55
Gallbladder wall	161 ± 2.52	158 ± 6.50	144 ± 3.06	143 ± 7.00
Left colon	154 ± 2.65	148 ± 4.50	121 ± 2.08	118 ± 3.50
Small intestine	145 ± 2.65	141 ± 4.00	121 ± 2.65	118 ± 3.00
Stomach wall	152 ± 2.31	146 ± 5.00	128 ± 1.73	124 ± 4.50
Right colon	149 ± 2.65	145 ± 4.00	124 ± 1.73	121 ± 4.00
Rectum	144 ± 2.65	140 ± 3.00	116 ± 2.08	113 ± 2.50
Heart wall	612 ± 13.1	526 ± 61.5	530 ± 10.7	473 ± 53.0
Kidneys	303 ± 12.0	288 ± 31.0	262 ± 10.5	250 ± 27.0
Liver	458 ± 34.0	495 ± 62.5	399 ± 30.0	432 ± 55.0
Lungs	150 ± 2.31	145 ± 5.50	121 ± 1.73	117 ± 4.50
Ovaries	451 ± 111	309 ± 116	–	–
Pancreas	162 ± 2.52	158 ± 6.00	132 ± 1.73	128 ± 5.00
Prostate	–	–	115 ± 2.08	112 ± 2.50
Salivary glands	131 ± 2.65	127 ± 3.00	110 ± 2.08	107 ± 2.50
Red marrow	112 ± 1.73	108 ± 3.50	93.0 ± 1.70	90.2 ± 2.65
Osteogenic cells	139 ± 2.65	135 ± 4.00	137 ± 2.65	133 ± 3.50
Spleen	416 ± 48.0	319 ± 45.5	357 ± 41.5	275 ± 39.5
Testes	–	–	106 ± 2.08	103 ± 2.50
Thymus	152 ± 2.65	146 ± 5.50	127 ± 2.08	122 ± 5.00
Thyroid	134 ± 2.65	130 ± 3.50	113 ± 2.52	109 ± 2.50
Urinary bladder wall	183 ± 6.24	182 ± 21.5	144 ± 4.73	139 ± 11.5
Uterus	145 ± 2.65	140 ± 3.00	–	–
Total body remainder	154 ± 2.31	149 ± 6.00	125 ± 1.73	122 ± 4.50
**Effective dose [μSv/MBq]**	**162 ± 6.11**	**153 ± 11.0**	**112 ± 1.15**	**109 ± 5.50**

IX. Fc receptor levels in myeloma cells

Fc receptor levels of U266, L363 myeloma cells and A375 melanoma cells

As shown in Figure S 11, we found no expression of the analyzed Fc receptors CD16, CD23, CD64, and CD32 in U266 cells. Of note, we detected a low expression of CD64 and marginal expression of CD23 in L363 cells as well as a marginal expression of CD32 in A375 cells. Therefore, we assume that the specific Fc receptors investigated herein play, if any, a very minor role in the accumulation of the radiolabeled anti-BCMA mAbs in U266, L363, and A375 xenograft tumors.

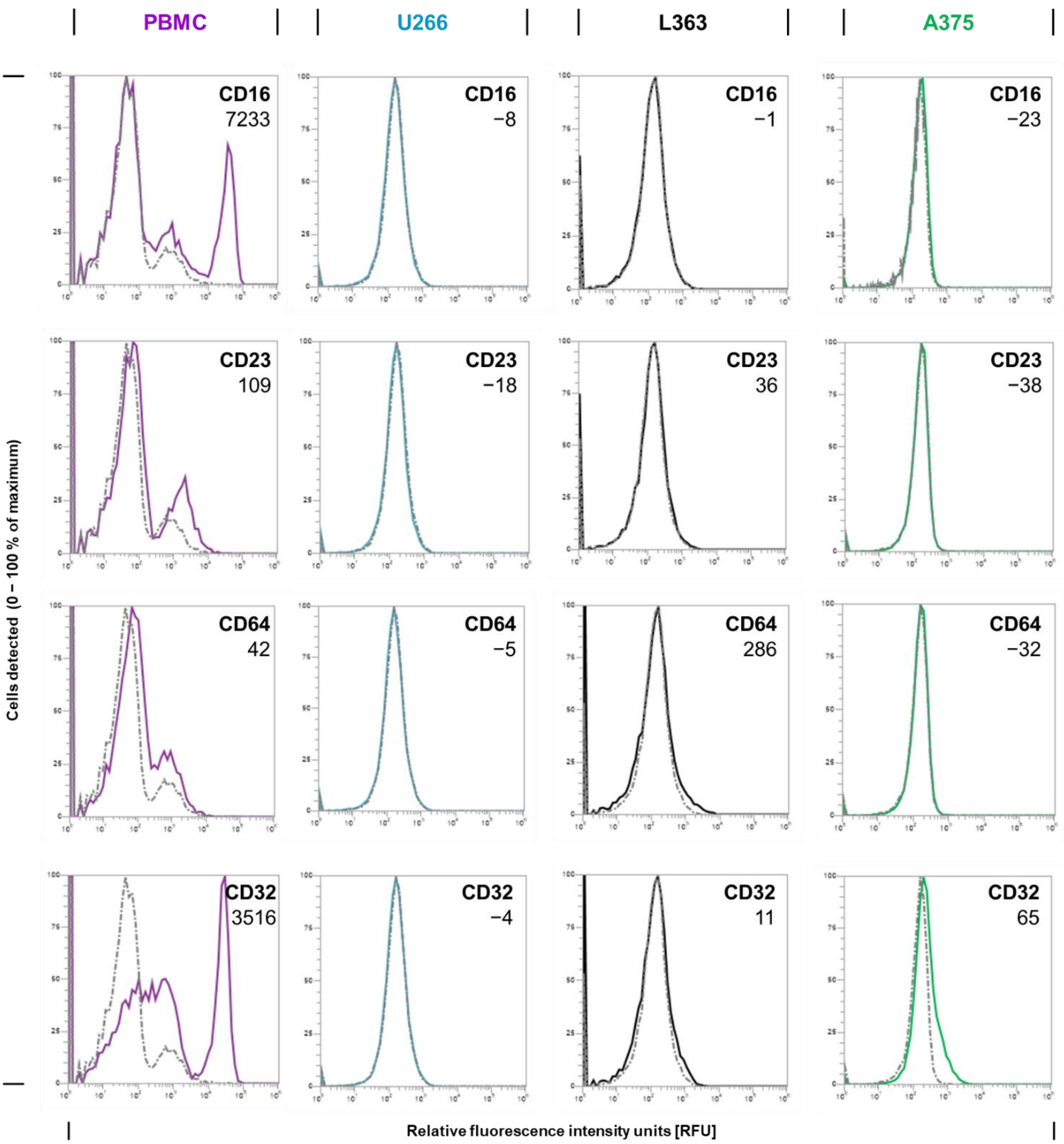

Figure S 11: Flow cytometry analysis of Fc receptors (CD16, CD23, CD64, CD32) in U266, L363, and A375 cells; (PBMC) human peripheral blood mononuclear cells serving as positive controls; (dash-dot line) negative control: respective cell sample measured without incubation with the primary antibody; For each cell sample, the Fc receptor-specific mean fluorescence intensity ratio (sample/negative control) is given

References

All references within the *Supporting information* are included in the main body of the article.
